# Shared features of cryptic plasmids from environmental and pathogenic *Francisella* species

**DOI:** 10.1371/journal.pone.0183554

**Published:** 2017-08-24

**Authors:** Jean F. Challacombe, Segaran Pillai, Cheryl R. Kuske

**Affiliations:** 1 Bioscience Division, Los Alamos National Laboratory, Los Alamos, New Mexico, United States of America; 2 Office of Laboratory Science and Safety, US Food and Drug Administration, Silver Spring, Maryland, United States of America; University of Manchester, UNITED KINGDOM

## Abstract

The *Francisella* genus includes several recognized species, additional potential species, and other representatives that inhabit a range of incredibly diverse ecological niches, but are not closely related to the named species. *Francisella* species have been obtained from a wide variety of clinical and environmental sources; documented species include highly virulent human and animal pathogens, fish pathogens, opportunistic human pathogens, tick endosymbionts, and free-living isolates inhabiting brackish water. While more than 120 *Francisella* genomes have been sequenced to date, only a few contain plasmids, and most of these appear to be cryptic, with unknown benefit to the host cell. We have identified several putative cryptic plasmids in the sequenced genomes of three *Francisella novicida* and *F*. *novicida*-like strains (TX07-6608, AZ06-7470, DPG_3A-IS) and two new *Francisella* species (*F*. *frigiditurris* CA97-1460 and *F*. *opportunistica* MA06-7296). These plasmids were compared to each other and to previously identified plasmids from other *Francisella* species. Some of the plasmids encoded functions potentially involved in replication, conjugal transfer and partitioning, environmental survival (transcriptional regulation, signaling, metabolism), and hypothetical proteins with no assignable functions. Genomic and phylogenetic comparisons of these new plasmids to the other known *Francisella* plasmids revealed some similarities that add to our understanding of the evolutionary relationships among the diverse *Francisella* species.

## Introduction

The *Francisella* genus is comprised of several recognized species, additional potential species, and outlier representatives that are not closely related to the named species [1–12]. *Francisella* species have been isolated from various clinical and environmental sources, and include highly virulent human and animal pathogens (*F*. *tularensis*), opportunistic human pathogens (*F*. *novicida*, *F*. *philomiragia*, *F*. *opportunistica* MA06-7296), fish pathogens (*F*. *noatunensis*), tick endosymbionts (*F*. *persica*), and potentially free-living isolates inhabiting seawater (*F*. *salina* TX07-7308, *F*. *uliginis* TX07-7310, *F*. *novicida* TX07-6608) and cooling systems (*Francisella* sp. W12-1067, *F*. *frigiditurris* CA97-1460, and *Allofrancisella guangzhouensis* [13]). Due to the diversity of environmental niches and limited genetic diversity among *Francisella* species, the taxonomic relationships among this genus have often been difficult to resolve [2–4, 6–19].

Only a few members of the *Francisella* genus carry plasmids; these include *F*. *novicida* strain F6168 [20, 21], *F*. *philomiragia* strains 25016, 25017, 25018, GA01-2794, GA01-2801 [22, 23], and *A*. *guangzhouensis* [13, 24]. Most of these *Francisella*-derived plasmids appear to be cryptic, with an unknown benefit, if any, to the host cell. Our previous work identified a large circular plasmid pFNPA10 in the genome of *F*. *novicida* strain PA10-7858 that was not closely related to other known plasmids [25]. We proposed that the pFNPA10 plasmid was unique to the *Francisella* genus, used the theta mode of replication, and was capable of conjugative transfer. Here, we identified putative plasmids in the genomes of the *F*. *novicida*-like strain TX07-6608 [15] isolated from seawater in the area of Galveston Bay, Houston, TX [18], *F*. *novicida* AZ06-7470 and *F*. *opportunistica* MA06-7296 isolated from human clinical samples [2, 26], *F*. *novicida* DPG_3A-IS from a warm spring [27], and *F*. *frigiditurris* CA97-1460 isolated from an air conditioning system [15]. The aim of this study was to characterize the sequences of these newly identified putative plasmid sequences, and compare them to each other and to the previously identified *Francisella* plasmids. We found that all of the plasmids were cryptic, encoding functions potentially involved in replication, conjugal transfer and partitioning, a few functions that could be important to environmental survival (transcriptional regulation, signaling, metabolic functions), and hypothetical proteins, to which a function could not be assigned. The plasmids from TX07-6608, AZ06-7470, DPG_3A-IS and CA97-1460 were somewhat similar to each other and to other *Francisella* plasmids, and comparison of their whole sequences, as well as phylogenetic analysis of replication proteins adds to our understanding of the evolutionary relationships among the *Francisella* species that carry plasmids.

## Materials and methods

For the genomes sequenced at Los Alamos National Laboratory (LANL), the bacterial cultivation, DNA extraction and annotation were performed as described previously (Table 1, [22, 27]). The actual sequencing methods varied somewhat for some of the genomes that were sequenced at LANL, so the details relevant to those genomes are presented here. For the *F*. *novicida* AZ06-7470 and *F*. *frigiditurris* CA97-1460 genomes, DNA was sequenced using Illumina [28] and PacBio [29] technologies. Illumina data were assembled together using Velvet, version 1.2.08 [30] and IDBA-UD, version 1.1.0 [31]. The PacBio data were assembled using HGAP, version 2.2.0 [32]. Consensus sequences from all assemblers were computationally shredded and merged using parallel Phrap, version SPS-4.24 [33, 34]. The resulting assembly was brought to improved status through both manual and computational finishing efforts using Consed [35] and in-house scripts. Assembled genome sequences were corrected by mapping Illumina reads (300X) back to the final consensus sequences using Burrows-Wheeler Alignment (BWA) [36], SAMtools [37] and in-house scripts. The final assembly of each genome consisted of one chromosome and one plasmid. The total length of the *F*. *novicida* AZ06-7470 genome was 1,925,251 bp, with average coverages of 366.66X and 338.86X for the Illumina and PacBio data, respectively. For the *F*. *frigiditurris* CA97-1460 genome, the total length was 1,861,609 bp with average coverages of 368.59X and 351.26X for the Illumina and PacBio data, respectively.

**Table 1:** Francisella plasmids.

Species	Plasmid	Size in bp (# ORFs)	GenBank Accession	Reference
Previously identified				
*Francisella philomiragia* ATCC25016 [O#319–029]*	pF242	3,936 (4)	NC_013091 [NZ_CP009342]	[22, 23]
*Francisella philomiragia* ATCC25017 [O#319–036]*	pF243 [pFPJ_1]	4,876 (7)	NC_013092 [NZ_CP009443]	[22, 23]
*Francisella philomiragia* ATCC25018 [O#319–067]*	pFPI_1	3,936 (4)	NZ_CP009437	[22]
*Francisella philomiragia* GA01-2794*	NA	4,016 (5)	NZ_CP009441	[22]
*Francisella philomiragia* GA01-2801*	pFPK_1	8,805 (8)	NZ_CP009446	[22]
*Francisella philomiragia* GA01-2801*	pFPK_2	2,402 (2)	NZ_CP009445	[22]
*Allofrancisella guangzhouensis* type strain 08HL01032	NA	3,045 (3)	NZ_CP010428	[13, 24]
*Francisella novicida* F6168	pFNL10	3,990 (6)	NC_004952	[21]
*Francisella novicida* PA10-7858*	pFNPA10	41,013 (57)	NC_023026	[25]
*Francisella novicida* DPG_3A-IS*	NA	41,959 (42)	NZ_CP010104	[27]
*Francisella hispaniensis* FSC454	pFSC454	16,037 (13)	NZ_CP018094	NA
*Francisella tularensis* subsp. tularensis strain SCHU S4 substr. NR-28534	NA	10,408 (10)#	NZ_CP010447	NA
*Francisella tularensis* subsp. tularensis strain SCHU S4 substr. NR-643	NA	3,195 (3)	NZ_KK211928	NA
*Francisella tularensis* subsp. tularensis strain SCHU S4 substr. NR-10492	NA	3,195 (3)	NZ_KK211930	NA
*Francisella tularensis* subsp. tularensis strain SCHU S4 substr. SL	NA	3,195 (3)	NZ_KK211926	NA
*Francisella tularensis* subsp. tularensis strain SCHU S4 substr. FSC043/FSC237	NA	3,195 (3)	NZ_KK211924	NA
New *Francisella* plasmids				
*Francisella novicida* TX07-6608*	plasmid 1	2,621 (1)	JRXS00000000	This paper
*Francisella novicida* TX07-6608*	plasmid 2	3,546 (3)	JRXS00000000	This paper
*Francisella novicida* TX07-6608*	plasmid 3	82,910 (91)	JRXS00000000	This paper
*Francisella novicida* TX07-6608*	plasmid 4	82,739 (102)	JRXS00000000	This paper
*Francisella novicida* AZ06-7470*	pFNE_1	34,471 (51)	CP009683	This paper
*Francisella frigiditurris* CA97-1460*	pFCD_1	6,175 (7)	CP009655	This paper
*Francisella opportunistica* MA06-7296*	NA	3,403 (5)	CP016929	This paper

^#^the plasmid from SCHU S4 substr. NR-28534 has 10 open reading frames representing potential coding sequences but 5 of them are annotated as pseudogenes

*Genomes sequenced (or re-sequenced) at Los Alamos National Laboratory

The *F*. *opportunistica* MA06-7296 genome sequence was generated using a combination of Illumina [28] and 454 technologies [38]. An Illumina GAii shotgun library was constructed and sequenced, generating 12,268,845 reads totaling 441.7 Mb; a 454 Titanium standard library generated 286,421 reads and two paired end 454 libraries with an average insert size of 7 Kb, and 9 Kb, which generated 99,600 reads totaling 90.9 Mb of 454 data. The 454 Titanium standard data and the 454 paired end data were assembled together with Newbler, version 2.3-PreRelease-6/30/2009. The Newbler consensus sequences were computationally shredded into 2 Kb overlapping fake reads (shreds). Illumina sequencing data was assembled with VELVET, version 1.0.13 [30], and the consensus sequences were computationally shredded into 1.5 Kb shreds. The 454 Newbler consensus shreds, the Illumina VELVET consensus shreds and the read pairs in the 454 paired end library were integrated using parallel phrap, version SPS—4.24 (High Performance Software, LLC, [33, 34]). Illumina data was used to correct potential base errors and increase consensus quality using the software Polisher developed at JGI (Alla Lapidus, unpublished). Possible mis-assemblies were corrected using gapResolution (Cliff Han, unpublished), or Dupfinisher [39]. The final assembly was based on 90.9 Mb of 454 draft data which provided an average 50.5X coverage of the genome and 441.7 Mb of Illumina draft data which provided an average 245.4X coverage of the genome.

For the *F*. *novicida*-like TX07-6608 genome, an Illumina short-insert paired-end library was constructed and sequenced, which generated 8,085,794 reads totaling 816.67 Mb. A PacBio long read library generated sub-reads totaling 510.58 Mb. Illumina data were assembled using Velvet, version 1.2.08 [30] and IDBA-UD, version 1.1.0 [31]. The PacBio data were assembled using HGAP, version 2.2.0 [32]. Consensus sequences from all assemblers were computationally shredded and merged using parallel Phrap, version SPS-4.24 [33, 34]. Possible mis-assemblies were corrected and some gap closure was accomplished with manual editing in Consed [33–35]. The final assembly was based on 533.23 Mb of Illumina data and 510.58 Mb of PacBio data to achieve 337.90X and 232.08X coverage of the genome, respectively.

All other plasmid sequences were obtained from GenBank. The plasmid sequences listed in Table 1 were aligned to each other using progressive Mauve [40]. Coding sequences from the new plasmids were used as queries in BLASTP searches [41] against the nr database to identify the closest hits in other bacterial genomes. To identify plasmid proteins with significant homologies within the *Francisella* genus, the predicted coding sequences from each plasmid were compared against each of the other plasmids and a complete set of *Francisella* genome sequences using BLASTP and TBLASTN with an E-value cutoff of 10^−5^. The web-based addgene plasmid analysis software (at http://www.addgene.org/analyze-sequence/) was used to identify restriction sites in the sequences of each of the plasmids. The OriFinder program [42] was used to identify DnaA boxes and Z-curves corresponding to AT and GC disparity. The default (*Escherichia coli*) DnaA box sequence was used for queries, since we could not find a *Francisella*-specific motif. GenSkew (http://genskew.csb.univie.ac.at) was used to compute the cumulative GC skew for each putative plasmid sequence. The Tandem Repeats Finder program [43] was used to identify direct (tandem) repeats (using parameters: 2 7 7 80 10 50 20) and Inverted Repeats Finder was used to identify inverted repeats [44] in each putative plasmid sequence. Circular maps of each plasmid were drawn using the CGView software [45], and additional labels (ori, ter, Rep, repeats, DnaA boxes, restriction site locations) were added to the maps manually. Additionally, the program CGView Comparison Tool [46] was used to compare groups of plasmids for coding sequence similarity.

Rep protein sequences were aligned by MUSCLE [47] within MEGA 7.0 [48], using default parameters. Maximum likelihood trees were constructed in MEGA using 500 bootstrap replicates [49] and the Jones-Taylor-Thornton (JTT) amino acid substitution model [50], assuming uniform substitution rates among all sites. The maximum likelihood heuristic method was nearest-neighbor interchange, the initial tree was neighbor-joining, and the branch swap filter was set to ‘very weak’ to perform more exhaustive optimization and explore a larger search space. The bootstrap consensus tree inferred from 500 replicates is taken to represent the evolutionary history of the taxa analyzed [51].

## Results

### Characteristics of putative *Francisella* plasmids

**Fig 1:**
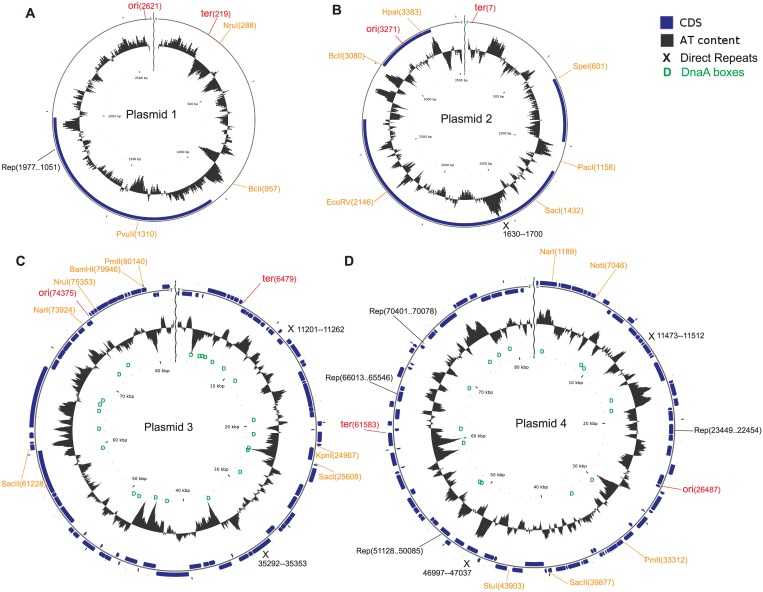
Circular maps of the candidate TX07-6608 plasmids. Maps were drawn by the CGView software (http://wishart.biology.ualberta.ca/cgview/index.html). Restriction sites were identified by the addgene software (http://www.addgene.org), and are indicated on the maps by orange annotation; ori and ter regions were calculated by the GenSkew program and their approximate locations are marked in red. Approximate locations of direct repeats are indicated by black Xs, and DnaA boxes by green Ds. Panel A. Plasmid 1. Panel B. Plasmid 2. Panel C. Plasmid 3. Panel D. Plasmid 4.

**Fig 2:**
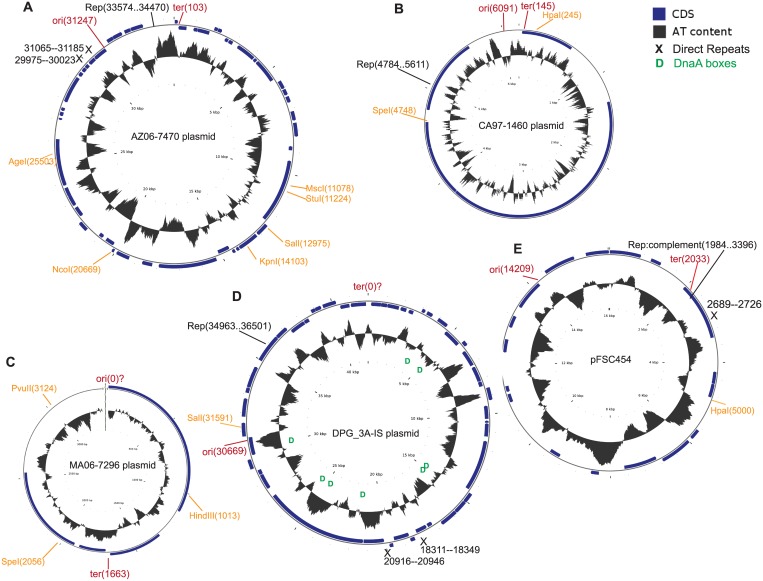
Circular maps of the (A) AZ06-7470, (B) CA97-1460, (C) MA06-7296, (D) DPG_3A-IS, and (E) pFSC454 plasmids. Maps were drawn by the CGView software (http://wishart.biology.ualberta.ca/cgview/index.html). Restriction sites were identified by the addgene software (http://www.addgene.org), and are indicated on the maps by orange annotation; ori and ter regions were calculated by the GenSkew program and their approximate locations are marked in red. Approximate locations of direct repeats and DnaA box clusters are indicated by black Xs and green Ds, respectively.

Putative plasmids were identified in the genome assemblies of four *Francisella* species. There were four extrachromosomal circular contigs in the *F*. *novicida* TX07-6608 genome assembly, ranging in size from 2,621 to 82,910 bp (Table 1, Fig 1). The genome assemblies of the other isolates each contained one extrachromosomal contig. In the *F*. *novicida* AZ06-7470 and *F*. *frigiditurris* CA97-1460 assemblies, the circular plasmid contigs had a size of 34,471 bp and 6,175 bp, respectively (Table 1, Fig 2). There was one extrachromosomal contig in *F*. *opportunistica* MA06-7296 with a size of 3,403 bp (Table 1). The *F*. *novicida* DPG_3A-IS genome contained one extrachromosomal contig with a size of 41,959 bp (Table 1). The topology of this plasmid, as well as the *F*. *hispaniensis* FSC454 plasmid, appeared to be circular (Fig 2, Panels D and E). A linear topology was suggested by the CGView software [45] for the putative plasmids from TX07-6608 and MA06-7296 (Figs 1 and 2).

### Analysis of putative plasmid sequences

The nucleotide sequences of the putative *Francisella* plasmids (Table 1) were aligned against each other using Progressive Mauve [40]. Likewise, the protein translations of each plasmid were aligned against each *Francisella* plasmid and against the nr database using BLASTP [41]. Supported by the top BLAST hits in S1 Table, Mauve alignments showed that *F*. *novicida* TX07-6608 plasmid 1, which contained only one protein coding region (for a Rep protein), had the largest region of nucleotide similarity with Rep-encoding regions in the named plasmids *F*. *philomiragia* GA01-2801 pFPK_2 and *F*. *philomiragia* ATCC 25016 pF242, and the plasmids from *A*. *guangzhouensis* 08HL01032 and *F*. *philomiragia* GA01-2794 (S1 Fig, Panel A). The Rep protein sequence from TX07-6608 plasmid 1 had 28%– 30% sequence identity with Rep proteins from these plasmids (S1 Table).

The TX07-6608 plasmid 2 had an overall nucleotide sequence arrangement similar to *F*. *novicida* F6168 plasmid pFNL10 (S1 Fig, Panel B) and had some regions in common with TX07-6608 plasmid 1. The TX07-6608 plasmid 2 also shared small regions of similarity with the *A*. *guangzhouensis* 08HL01032 plasmid. In particular, a helix-turn-helix domain protein (KX00_2304) had 68% amino acid sequence identity to a similar protein in the *A*. *guangzhouensis* 08HL01032 plasmid (S1 Table). Other small regions were similar to *F*. *philomiragia* ATCC 25017 [O#319–067] plasmid pF243/pFPJ_1, and the plasmid from *F*. *philomiragia* GA01-2794. The TX07-6608 plasmids 3 and 4 were most similar to each other and each had regions in common with plasmid pFNPA10 from *F novicida* PA10-7858 [25], and the plasmids from *F*. *novicida* strains AZ06-7470 and DPG_3A-IS (S2 Fig). The plasmid from *F*. *hispaniensis* FSC454 had three small regions of similarity to the DPG_3A-IS plasmid (S2 Fig). The *F*. *opportunistica* MA06-7296 plasmid had only one small region of similarity to pFPK_1 from *F*. *philomiragia* GA01-2801 (S1 Fig, Panel C). The *F*. *frigiditurris* CA97-1460 plasmid did not show any significant blocks of nucleotide similarity in Mauve alignments with the other *Francisella* plasmids (data not shown).

To better characterize each of the putative plasmids from TX07-6608, MA06-7296, AZ06-7470, CA97-1460, DPG_3A-IS and FSC454, we compared their protein coding features to the known protein sequences in GenBank and to the coding sequences from each of the other *Francisella* plasmids. S1 Table shows all of the features of the small plasmids, and only the non-hypothetical protein features of the larger plasmids, which included putative replication initiation proteins, mobile elements, conjugal transfer proteins, DNA-binding proteins, plasmid partitioning proteins, transcriptional regulators and group II introns. The TX07-6608 plasmids 1 and 2 were small, having only one and three ORFs, respectively. TX07-6608 plasmids 3 and 4 were larger and contained a similar functional repertoire of protein coding sequences, including putative mobile elements, transcriptional regulators, partitioning proteins, DNA binding proteins, group II intron reverse transcriptases and conjugal transfer proteins. In particular, plasmid 3 had nineteen genes that potentially encode transposases, four genes for DNA binding proteins, five genes encoding group II intron reverse transcriptases, two genes encoding putative ParA/ParB partitioning proteins and four genes encoding conjugal transfer proteins (TraA, TraF, 2 TraG). Plasmid 4 had forty genes encoding putative integrases/transposases, two genes encoding DNA binding proteins (HU), three genes for group II intron reverse transcriptases, and one gene each encoding ParM, ParB and TraA homologs.

**Fig 3:**
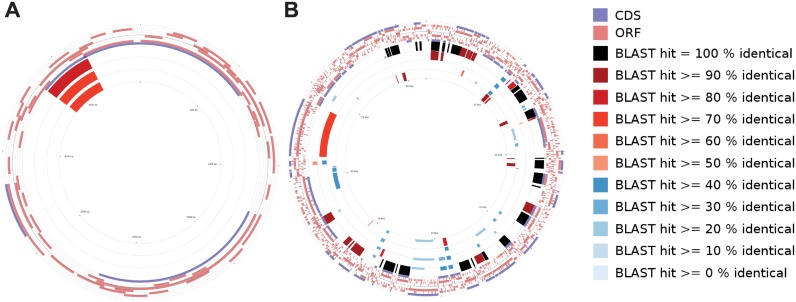
Plasmid maps drawn with the CGView comparison tool. Panel A. *F*. *philomiragia* 25016 plasmid pF242 was used as the reference and compared to *F*. *philomiragia* GA01-2801 pFPK_2, the plasmid from *A*. *guangzhouensis*, and the *F*. *philomiragia* GA01-2794 plasmid. The outermost ring shows the coding sequences of the reference, the pink rings moving toward the center show the ORFs of the comparison plasmids (in the order pFPK_2, *A*. *guangzhouensis*, GA01-2794), followed by the reverse strand coding sequences of the pF242 reference. The inner rings represent BLAST hits of the reference coding sequences to each other plasmid in the order listed above. Panel B. TX07-6608 plasmid 3 was used as the reference for comparison to TX07-6608 plasmid 4, the DPG_3A-IS plasmid, pFNPA10, and the plasmid from AZ06-7470, with the rings representing the ORFs in this order from the outer edge toward the center. The tool will only show the ORFs from up to three comparison plasmids, so the ORFs from the AZ06-7470 plasmid were not included in the figure. However, the blast comparison rings are shown for all four of the comparisons, in the order listed above. The parameters for the BLAST comparisons were: minimum ORF length = 25, expect value = 0.1, minimum score = 25, number of hits to keep for each query = 50, minimum hit proportion (query coverage) = 0.3.

Of particular interest was the gene content of each plasmid and how much of it was conserved from plasmid to plasmid. To assess plasmid gene content and homology, we used the CGView comparison tool [46], which employs BLAST to compare coding sequences and provides a circular map display for visual comparison. Results of this analysis were obtained for two groups of plasmids (Fig 3). The plasmids in each group were chosen based in their similarities to each other, determined by the Mauve analysis (S1 and S2 Figs). Fig 3 (Panel A) shows the *F*. *philomiragia* GA01-2801 pFPK_2, the plasmid from *A*. *guangzhouensis*, and the *F*. *philomiragia* GA01-2794 plasmid compared to *F*. *philomiragia* 25016 plasmid pF242. The one region of blast similarity indicates a partial alignment of the putative Rep proteins in each the plasmids. In Fig 3 (Panel B), TX07-6608 plasmid 4, the DPG_3A-IS plasmid, pFNPA10, and the plasmid from AZ06-7470 are compared to TX07-6608 plasmid 3. TX07-6608 plasmids 3 and 4 shared the most content, but all of the plasmids showed regions of similar content when compared to each other. The other plasmids (not in either group) showed no BLAST similarity to other plasmids by this analysis (not shown).

To compare putative Rep protein sequences among the *Francisella* plasmids, BLAST analysis was performed using as queries the Rep protein sequences identified in the *F*. *novicida* plasmids pFNPA10, pFNL10, TX07-6608 plasmids 1 and 4, the *F*. *philomiragia* GA01-2974 plasmid, *F*. *philomiragia* plasmids pFPK_1, pFPK_2, pFPI_1, the *A*. *guangzhouensis* plasmid, and the plasmids from *F*. *novicida* DPG_3A-IS and *F*. *hispaniensis* FSC454. This analysis showed that the putative Rep protein from TX07-6608 plasmid 1 had only ~30% identity to Rep-1 from the *F*. *philomiragia* and *A*. *guangzhouensis* plasmids. TX07-6608 plasmid 2 had three ORFS and did not have any genes encoding known replication proteins. The TX07-6608 plasmid 3 did not have any obvious genes encoding replication proteins, and BLASTP/TBLASTN of the Rep protein sequences from the other Francisella plasmids did not identify any by sequence similarity. However, this plasmid did have three genes encoding putative single-stranded DNA-binding proteins (KX00-2122, KX00-2136, KX00-2149), which could be involved in replication. TX07-6608 plasmid 4 had several genes encoding initiator replication protein homologs (KX00-2231, KX00-2266, KX00-2285, KX00-2291), although two of these (KX00-2285, KX00-2291) were of shorter length and only aligned partially with Rep sequences from the other Francisella plasmids. KX00-2285 aligned with the N-terminal of Rep query sequences, while KX00-2291 aligned with the C-terminal region of the query sequences, suggesting that they may once have been full length Rep sequences.

The original annotation of the AZ06-7470 plasmid included fifty-one coding sequences, but we found a putative RepB-encoding sequence near the origin that was not present in the original annotation (S1 Table, Fig 2 Panel A). More than half of the coding sequences encoded hypothetical proteins with no significant similarity to any known proteins. This plasmid additionally encoded fifteen potential mobile elements, two regulators, a restriction-modification methylase, and a putative partitioning protein, ParA.

As listed in S1 Table, two of the coding sequences from the MA06-7296 plasmid were most similar to a plasmid recombination enzyme (63%) and a hypothetical protein (94%) from *Clostridium botulinum*. The other three coding sequences did not have sequence similarity to any known proteins. This plasmid did not contain an obvious Rep encoding gene. The CA97-1460 plasmid (S1 Table, Fig 2 Panel B) had seven protein coding sequences, but only one of them, encoding a putative RepB, had similarity to the other *Francisella* plasmids. The RepB sequence from the CA97-1460 plasmid had 43% amino acid identity to RepB from TX07-6608 plasmid 4, only partially aligned with Rep from pFNPA10 (54% identity) and had 35% identity to RepB from the DPG_3A-IS plasmid. It was even less similar to RepB from the *F*. *philomiragia* plasmids (ranging from 0 to 22% amino acid identity, not shown). The previously sequenced plasmids from *F*. *novicida* DPG_3A-IS and F. *hispaniensis* FSC454 were included in this study for comparison purposes. Each of these plasmids contained protein coding sequences with similarity to pFNPA10 from *F*. *novicida* PA10-7858, and plasmids 3 and 4 from *F*. *novicida*-like TX07-6608 (S1 Table).

### Phylogenetic analysis of putative Rep protein sequences

**Fig 4:**
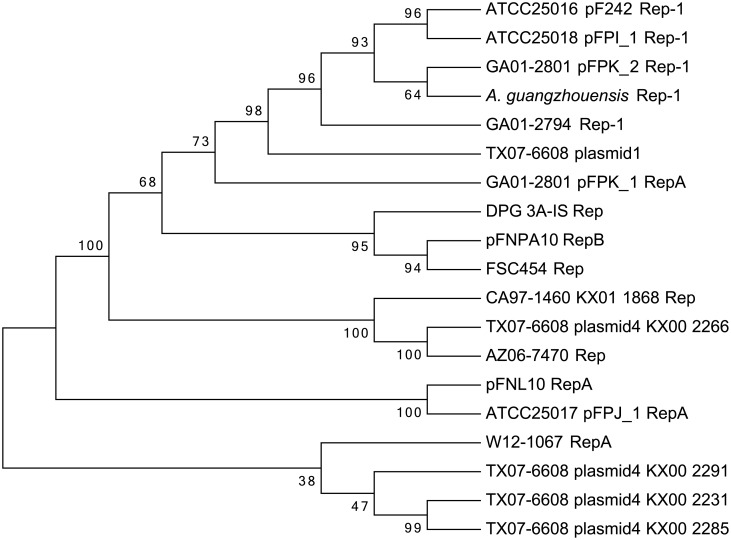
Phylogenetic analysis of putative Rep protein sequences. Evolutionary history was inferred by using the Maximum Likelihood method based on the JTT matrix-based model. The bootstrap consensus tree inferred from 500 replicates represents the evolutionary history of the taxa analyzed. The percentage of replicate trees in which the associated taxa clustered together in the bootstrap test (500 replicates) are shown next to the branches. Initial tree(s) for the heuristic search were obtained automatically by applying Neighbor-Join and BioNJ algorithms to a matrix of pairwise distances estimated using a JTT model, and then selecting the topology with superior log likelihood value. This analysis involved 19 amino acid sequences. There were a total of 551 positions in the final dataset. The Rep protein sequences KX00_2285 and KX00_2291, from TX07-6608 plasmid 4, were partial sequences.

Phylogenetic analysis of putative Rep protein sequences (Fig 4) revealed relationships similar to those identified by Mauve nucleotide alignments and the BLASTP analyses (S1 Table). Three of the Rep sequences from TX07-6608 plasmid 4 (KX00_2231, KX00_2285, KX00_2291) were most similar to each other (47% and 99% branch support values). The *Francisella* sp. W12-1067 genome had a putative Rep encoding gene and the predicted protein sequence was most closely related to the three Rep sequences from TX07-6608 plasmid 4 (38% support). The Rep sequence from *F*. *novicida* F6168 plasmid pFNL10 was most closely related to that from *F*. *philomiragia* ATCC25017 plasmid pFPJ_1 (100% branch support). The other potential Rep protein from TX07-6608 plasmid 4 (KX00-2266) was in the same minor branch as Rep from *F*. *novicida* AZ06-7470 (100%) and the Rep sequence from CA97-1460 was related to these with 100% branch support. The prospective Rep protein from TX07-6608 plasmid 1 was in the same major group as Rep from *F*. *philomiragia* GA01-2794, pF242, pFPI_1, pFPK_2 and *F*. *guangzhouensis* (98% branch support). The RepB sequence from *F*. *novicida*-like PA10-7858 plasmid pFNPA10 was most closely related to the putative Rep from *F*. *hispaniensis* FSC454 (94%), and these were in in the same clade with RepB from *F*. *novicida* DPG_3A-IS (95% branch support).

### Replication-related features

In addition to Rep genes, other replication-related features may indicate an origin of replication in a bacterial chromosome or plasmid; these include high AT content, the presence of restriction sites, and repeated sequences, which may indicate DnaA boxes, as well as 13 nucleotide-long motifs (tandem repeats) (reviewed by [52, 53]). AT rich regions can be identified by visualizing the GC skew. The GenSkew program (http://genskew.csb.univie.ac.at/) calculates the normal and cumulative GC skew by sliding a window over a given sequence. Given the number of Gs and Cs in the sequence, the skew is calculated as G − C/G + C. The cumulative graph adds up the values for all previous windows up to the current position, and displays the global minimum and maximum GC skew, which be used to predict the origin of replication (minimum) and the terminus location (maximum) in prokaryotic genomes. Calculation of the cumulative GC skew using the GenSkew program showed a potential origin and terminus of replication in each plasmid sequence (Table 2, S3 and S4 Figs), except the MA06-7296 plasmid, for which we did not find an ori (Fig 2, Panel C), and the DPG_3A-IS plasmid, which had a maximum at 0 but this was not indicated as a potential terminus on the plot (S4 Fig).

**Table 2:** Coordinates of origin and terminus of replication of TX07-6608, AZ06-7470, CA97-1460 and MA06-7296 plasmids.

Sequence	Origin (minimum GC skew)	Terminus (maximum GC skew)
TX07-6608 plasmid1*	2,621	219
TX07-6608 plasmid2*	3,271	7
TX07-6608 plasmid3*	74,375	6,479
TX07-6608 plasmid4*	26,487	61,583
AZ06-7470	31,247	103
CA97-1460	6,091	145
MA06-7296*	0	1,663
DPG_3A-IS	30,669	0
pFSC454	14,209	2,033

*appears to be a linear plasmid

The addgene program identified three restriction sites (for NruI, BcII and PvuII) in the sequence of TX07-6608 plasmid 1 (Fig 1, Panel A). However, the OriFinder tool would not process the sequence for identification of DnaA boxes, and Tandem Repeats Finder did not find any direct repeats. Plasmid 2 (Fig 1, Panel B) had six restriction sites, and one region identified by Tandem Repeats Finder that contained 5.4 copies of a 12-mer repeat (identified by an ‘X’ in the figure). OriFinder would not process the plasmid 2 sequence. For plasmids 2, 3 and 4, the Tandem Repeats Finder output is listed in S2 Table. Plasmid 3 (Fig 1, Panel C) had seven restriction sites, and two regions identified by Tandem Repeats Finder; each region contained 5.2 copies of a 12-mer repeat. Plasmid 4 (Fig 1, PanelD) had five restriction sites and two regions of direct repeats, the first repeat region had three copies of a 13-mer repeat and the second region had two copies of a 20-mer repeat. Plasmids 3 and 4 each contained numerous potential DnaA boxes, as identified by OriFinder (Fig 1, S5 Fig). However, OriFinder did not identify a possible origin of replication in either plasmid sequence. Because OriFinder did not process plasmids 1 and 2, we searched the sequences of these plasmids for the DnaA box sequences identified in plasmids 3 and 4, but we did not identify any DnaA boxes in plasmids 1 and 2 by this method.

The AZ06-7470 plasmid had six restriction sites and two regions containing repeat motifs (Fig 2). The CA97-1460 plasmid had two restriction sites, the MA06-7296 plasmid had three, and the DPG_3A-IS plasmid and pFSC454 each had one (Fig 2). Of these latter three putative plasmids, OriFinder would only process the DPG_3A-IS sequence (S5 Fig), and therefore we did not identify any DnaA boxes in the others. Tandem Repeats Finder identified two regions containing repeat motifs in the DPG_3A-IS plasmid, one in pFSC454 (S2 Table), but none in the plasmids from MA06-7296 and CA97-1460. The first repeat region in AZ06-7470 had 6.1 copies of an 8-mer repeat, while the second region had 15.1 copies of a different 8-mer repeat. Both of these repeat regions were located close to the ori region of this plasmid (Fig 2, Panel A). The DPG_3A-IS plasmid had 2.2 copies of an 18-mer repeat and 3.4 copies of a 9-mer repeat, while pFSC454 had 2.1 copies of an 18-mer repeat. None of the repeats were near the origins of these two plasmids. OriFinder identified nine dnaA box clusters in the DPG_3A-IS plasmid sequence, and one of these was near the putative origin (Table 2, Fig 2, S5 Fig).

### Coding sequence similarities among Francisella plasmids

The plasmid from *F*. *novicida* DPG_3A-IS showed some small regions of similarity with TX07-6608 plasmids 3 and 4, as well as with pFNPA10 and the plasmid from AZ06-7470 (S2 Fig, S3 Table). This plasmid had eight predicted coding sequences in common with pFNPA10, including RepB, fifteen that were similar to TX07-6608 plasmid 3, eleven in common with TX07-6608 plasmid 4, including RepB, one in common with the AZ06-7470 plasmid and only RepB in common with the CA97-1460 plasmid (S3 Table). The plasmid from Schu S4 substr. NR-28534 had only five potential coding sequences, with no similarity (via BLASTP analysis) to the coding sequences from the other *Francisella* plasmids (S4 Table).

## Discussion

Bacterial plasmids are genetic elements that can exist outside of the chromosome. Plasmids usually carry at least one expressed gene, and typically require chromosomally encoded components for replication [52–54]. Plasmids can carry traits beneficial to host cells, for example antibiotic or heavy metal resistance, virulence factors or specific metabolic functions that enhance the survival of host cells and influence bacterial evolution [55]. However, some plasmids are cryptic, with largely unknown functions and no obvious benefit to the host cells that carry them [56].

Previously, only two *Francisella* species (*F*. *novicida*, *F*. *philomiragia*) were shown to carry plasmids (Table 1), and most of these appeared to be cryptic, mainly encoding proteins with putative functions in plasmid replication and maintenance [21, 23, 25]. Here we characterized four contigs, representing putative plasmids, in the assembled genome of the *F*. *novicida*-like strain TX07-6608, which was isolated from seawater in the area of Galveston Bay, Houston, TX [18], and a single plasmid in the each of the genomes of *F*. *opportunistica* MA06-7296 and *F*. *novicida* AZ06-7470, isolated from human clinical samples [2, 26, 57] and *F*. *frigiditurris* CA97-1460 cultured from an air conditioning system. Analysis of these plasmids revealed that they too appear to be cryptic, encoding a few functions potentially involved in replication, conjugal transfer and partitioning. Comparison of the *Francisella* plasmids revealed some similarities among them. However, none of the plasmids were completely syntenic.

Functional self-replicating plasmids generally contain one or more origins of replication, at least one regulatory element, and a primase protein (such as Rep) to initiate replication [55, 58]. Depending on the mode of replication employed, a plasmid may contain direct repeats and an AT-rich region near the origin of replication. While experimentation is necessary to determine whether any of the plasmids presented here are capable of replication and persistence in host cells, we did identify replication-associated features in each of the plasmids. Potential replication origin and termination sites were found by examining AT rich regions and GC-Skew (S3 and S4 Figs). Potential DnaA binding sites (boxes) were present in some of the plasmid sequences (Figs 1 and 2, S5 Fig). However, the presence of DnaA boxes is not a universal feature of replication origins, particularly in plasmids; instead, the most conserved structural feature is an AT-rich region [52, 53], which often contains tandem direct repeats [52]. While AT-rich tandem repeats were present in TX07-6608 plasmids 2–4, the DPG_3A-IS plasmid, and pFSC454, none of them were co-located with the putative ori region (Figs 1 and 2). However, the tandem repeats in the AZ06-7470 plasmid were located near the ori region (Fig 2).

Due to the presence of Rep-encoding genes, and the lack of obvious iteron-like repeats in their ori regions, TX07-6608 plasmid 1 and the CA97-1460 plasmid might replicate via the theta or rolling circle mechanisms [59], as they are small (< 10 Kb) and rolling circle replication is usually confined to such small plasmids [60]. The TX07-6608 plasmid 4, the DPG_3A-IS plasmid and pFSC454 were each greater than 10Kb in size and contained putative Rep-encoding genes, so they might be theta-replicating plasmids. Previous work demonstrated that *F*. *philomiragia* plasmid pF243 is a theta-replicating plasmid similar to the plasmid pFNL10 from *F*. *novicida*-like strain F6168 [23]. Likewise, the pFNPA10 plasmid from *F*. *novicida*-like strain PA10-7858 contained iteron-like direct repeats and an ORF encoding a putative replication protein, suggesting the theta mode of replication [25]. Because it contained iteron-like direct repeats near the origin and a replication protein coding sequence, the *F*. *novicida* AZ06-7470 plasmid may also replicate via the theta mechanism.

TX07-6608 plasmids 2 and 3 did not encode any apparent Rep proteins, direct repeats were not located in the putative ori regions, and plasmid 2 did not contain any likely DnaA boxes, although plasmid 3 did. The CA97-1260 and MA06-7296 plasmids were also in this situation. The absence of a plasmid-encoded Rep protein potentially rules out self-replication. However, plasmids do not always encode every function required for replication, and it is possible that these plasmids are dependent on replication enzymes encoded on the other plasmids or on the host cell chromosome. For example, there are small plasmids, such as ColE1 and R1 [54, 61], which do not encode any replication functions, and rely on plasmid-encoded RNA species as well as host-encoded proteins for replication in *Escherichia coli*. Plasmids like ColE1 require the enzymes DNA polymerase I, DNA-dependent RNA polymerase, and DNA polymerase III [54], which are all encoded by the TX07-6608, AZ06-7470, CA97-1460 and MA06-7296 chromosomes, along with DnaA, PriA, and DNA gyrase (data not shown; see NCBI accession numbers JRXS00000000, CP009682, CP009654 and CP016929)

Some plasmids, termed conjugative plasmids, are transmissible by conjugation, a horizontal transfer mechanism that facilitates the spread of genes among bacteria and contributes to a dynamic gene pool in microbial communities [62]. Conjugative plasmids can carry accessory genes that contribute adaptive traits to their hosts and provide the means to respond to environmental stress, adapt within specific environmental niches, and colonize new niches [63]. Conjugative plasmids have a core backbone, which contains elements required for replication, maintenance, stability and conjugative transfer, and a flexible set of accessory genes, which provide the adaptive traits (reviewed by [63]).

Conjugative plasmids must have an oriT region, and genes encoding a DNA relaxase, a type 4 coupling protein, and a type 4 secretion system (reviewed by [64]) which delivers plasmid DNA to the host cell [65]. DNA relaxase binds to the oriT region and is essential to the initiation and termination of conjugative plasmid transfer [66]. Non-conjugative plasmids do not encode a DNA relaxase, so are incapable of initiating conjugation, but they can be transferred with the assistance of conjugative plasmids. An intermediate class of mobilizable plasmids carry only a subset of the genes required for transfer: a DNA relaxase and oriT. Some mobilizable plasmids also encode a type 4 coupling protein [66].

The TX07-6608 plasmids 3 and 4 encoded a partial set of putative conjugative transfer proteins; Plasmid 3 encoded TraA, TraF and 2 copies of TraG, while plasmid 4 encoded TraA. TraA is a relaxase [67], while TraG functions as an NTP hydrolase and also as a component of type IV secretion systems [68], and is essential for DNA transfer in bacterial conjugation. There is evidence that TraG-like proteins couple the relaxosome to the DNA transport machinery [69] and that this may occur because TraG forms a channel through which single stranded DNA can pass [68]. TraF is a periplasmic membrane protein component that spans the Gram-negative cell membrane and is part of a type IV secretion system [70]. Since these two plasmids seemed like they could be mobilizable, we tried to identify the oriT region, which TraA would bind to in order to initiate plasmid transfer. Since the oriT regions of conjugative and mobilizable plasmids often contain inverted repeats [71, 72], we used the Inverted Repeats Finder program [44] to try to identify inverted repeats and a putative oriT region. As recommended by the authors of the tool, we tried several different parameter sets, including Parameters: 2 3 5 80 10 40 100000 500000, Parameters: 2 3 5 80 10 40 10000 10000 -d -t4 74 -t5 493 -t7 10000, and Parameters: 2 3 5 80 10 40 500000 10000 -d -h -t4 74 -t5 493 -t7 10000. However, we were unable to identify inverted repeats in any of the plasmids. TX07-6608 plasmids 3 and 4 each had a coding sequence with similarity to type I plasmid partition protein ParB. TX07-6608 plasmids 3 and 4 each had one coding sequence next to their version of ParB, with similarity to ParA from W12-1067 (S1 Table). The plasmid from *F*. *novicida* AZ06-7470 also had a gene encoding a putative ParA. As both ParA and ParB are necessary for directed plasmid partitioning during cell division, it is possible that these plasmids have this function [73]. The plasmid from *F novicida* DPG_3A-IS had one gene encoding the type II plasmid partition protein ParM (analogous to ParA) and two genes encoding the cell division protein Fic. This plasmid was lacking a gene for ParR, which is analogous to ParB. None of the plasmids had a gene encoding ParC, which is apparently needed for a complete partitioning system.

The only function encoded in the MA06-7296 plasmid was a mobilization protein/plasmid recombination enzyme with 63% sequence similarity to a plasmid recombination enzyme from *C*. *botulinum*. The CGView software suggested a linear topology for this plasmid, and we could not identify an *ori* region, indicating that this plasmid may truly be a linear replicon, or the sequence may not be complete. The CA97-1460 plasmid also encoded a mobilization protein (MobB). An additional interesting finding is that the genome of W12-1067 included RepA and Phd and YoeB/Doc toxin-antitoxin proteins, which were also present in pFPJ_1, pF243 and pFNL10 (data not shown). Since this genome is draft quality, it was not possible to determine synteny with the other plasmids. The coding sequences in W12-1067 that showed some similarity to the above mentioned *Francisella* plasmids were not all present in one contig. In fact, some of them were found in larger contigs, so whether or not W12-1067 contains a separate plasmid replicon or an integrated plasmid, or various chromosomal sequences of plasmid origin remains to be determined.

An important, yet unresolved question about cryptic bacterial plasmids has focused on whether or not they are stably maintained in bacterial communities, since they impose a metabolic cost to the host but confer no obvious advantage. A recent study described the isolation and characterization of a diverse set of cryptic plasmids from different freshwater sources that were not under strong selection (i.e., not from polluted soil or water, from wastewater treatment plants or from pathogen cultures) [74]. Some of the plasmids that were isolated and sequenced carried only core genes involved in plasmid functions, suggesting that cryptic plasmids may persist in natural environments [74]. Our results suggest that this may also be the case for the cryptic plasmids carried by environmental and clinical *Francisella* species. However, their specific roles and whether or not the coding sequences that lack a functional definition may provide a potential benefit to their host cells remain to be determined.

## Conclusions

While bacterial plasmids can carry traits that enhance the survival of host cells and influence bacterial evolution [55], cryptic plasmids encode few functions other than those needed to replicate and mobilize. With no obvious benefit to the host cells that carry them [56], cryptic plasmids are somewhat of an enigma. While cryptic plasmids have been shown to persist in natural environments [74], our results comparing the cryptic plasmids in diverse *Francisella* genomes show that they are also found in clinical isolates. These results provide a new understanding of the phenotypic variability and complex taxonomic relationships among the known *Francisella* species, and also give us new plasmid features to use in characterizing related species groups. However, there are still many cultured *Francisella* isolates for which we still have no genomic sequence; it will only be through the sequencing and comparison of many more environmental and near neighbor *Francisella* isolates that we will be able to identify genomic features that enable us to accurately discriminate the various species groups.

## Supporting information

S1 Fig Progressive Mauve nucleotide sequence alignments of F. novicida-like strain TX07-6608 plasmids 1 and 2, and the plasmid from *Francisella* sp. MA06-7296 with the other *Francisella* plasmids that were most similar. Regions of similarity in the comparisons are shown in green and red.
(PDF)

S2 Fig Progressive Mauve alignment of the AZ06-7470 plasmid with TX07-6608 plasmids 3 and 4, pFNPA10 from *F*. *novicida*-like strain PA10-7858, the DPG_3A-IS plasmid, and the pFSC454 plasmid, which were the most similar. The different regions of similarity are shown in different colors.
(PDF)

S3 Fig Cumulative GC skew plots for the TX07-6608 plasmids, as generated by the GenSkew program. The potential ori and ter regions are indicated by yellow vertical lines at the minimum and maximum GC skew values. Panel A. Plasmid 1. Panel B. Plasmid 2. Panel C. Plasmid 3. Panel D. Plasmid 4.
(PDF)

S4 Fig Cumulative GC skew plots for the plasmids from AZ06-7470, CA97-1460, MA06-7296, DPG_3A-IS and FSC454. The potential ori and ter regions are indicated by yellow vertical lines at the minimum and maximum GC skew values. Panel A. AZ06-7470 plasmid. Panel B. CA97-1460 plasmid. Panel C. MA06-7296 plasmid. Panel D. DPG_3A-IS. Panel E. FSC454. The MA06-7296 plasmid did not have an ori region identified by this analysis, but the minimum GC skew value near 0. The DPG_3A-IS plasmid did not have a ter region identified by this analysis, by a maximum GC slew value occurred near 0.
(PDF)

S5 Fig Z-curves (AT, GC, RY and MK disparity curves) from OriFinder analysis of TX07-6608 plasmids 3 (Panel A) and 4 (Panel B), and the DPG_3A-IS plasmid (Panel C). Purple peaks with diamonds indicate the DnaA box clusters.
(PDF)

S1 Table Features of the *Francisella novicida-like* TX07-6608, *F*. *opportunistica* MA06-7296, *F*. *novicida* AZ06-7470, *F*. *frigiditurris* CA97-1460, F. novicida DPG_3A-IS, and F. hispaniensis FSC454 plasmids in comparison to all known *Francisella* plasmids and to the NCBI nr database. (PDF)

S2 Table Tandem Repeats Finder output for the plasmids that had direct repeats. (PDF)

S3 Table Comparison of the coding sequences from the *F*. *novicida* DPG_3A-IS plasmid to all known *Francisella* plasmids. (XLSX)

S4 Table Comparison of the coding sequences from the *F*. *tularensis* Schu S4 substr. NR-28534 plasmid to all known *Francisella* plasmids.
(XLSX)
