# Supplementary material for: Shared features of cryptic plasmids from environmental and pathogenic Francisella species
Source: PLoS One. 2017 Aug 24;12(8):e0183554. doi: 10.1371/journal.pone.0183554 (PMC5570271; doi:10.1371/journal.pone.0183554)

Figure S1. Progressive Mauve nucleotide sequence alignments of *F. novicida*-like strain TX07-6608 plasmids 1 and 2, and the plasmid from *Francisella* sp. MA06-7296 with the other Francisella plasmids that were most similar. Regions of similarity in the comparisons are shown in green and red.

A. TX07-6608 plasmid 1 was most similar to *F. philomiragia* plasmids pFPK_2, pF242, the GA01-2794 plasmid and the plasmid from *A. guangzhouensis.*

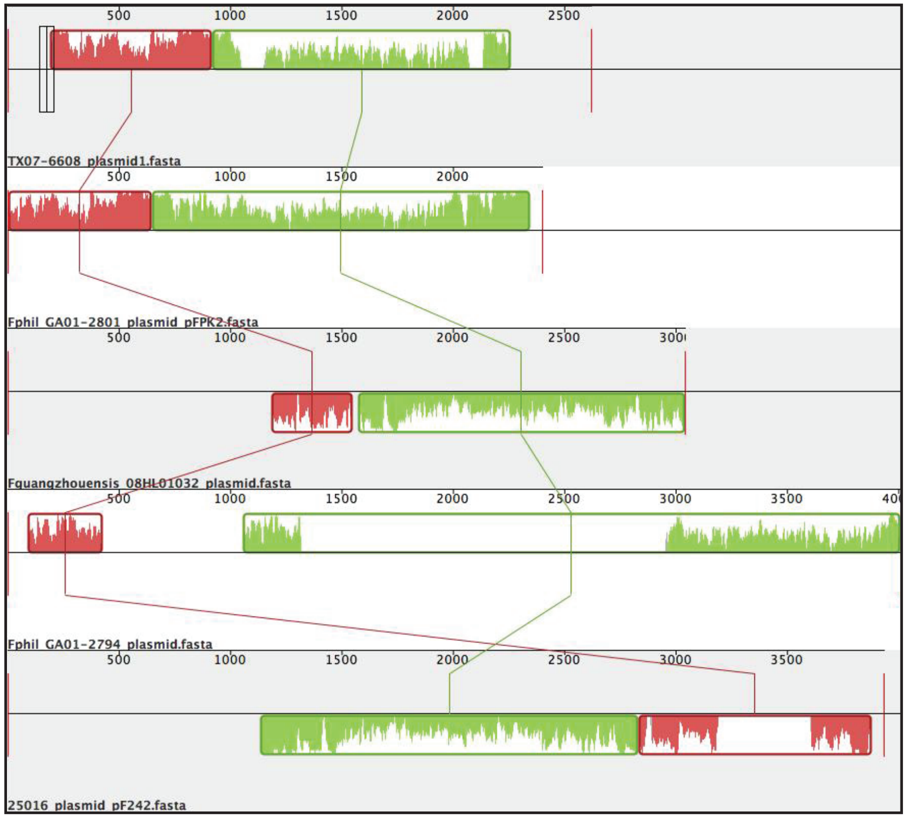

B. TX07-6608 plasmid 2 was most similar to pFNL10 from *F. novicida*-like strain F6168, and had smaller regions in common with other Francisella plasmids (*A. guangzhouensis* plasmid, TX07-6608 plasmid 1, pFPJ_1, and the GA01-2794 plasmid).

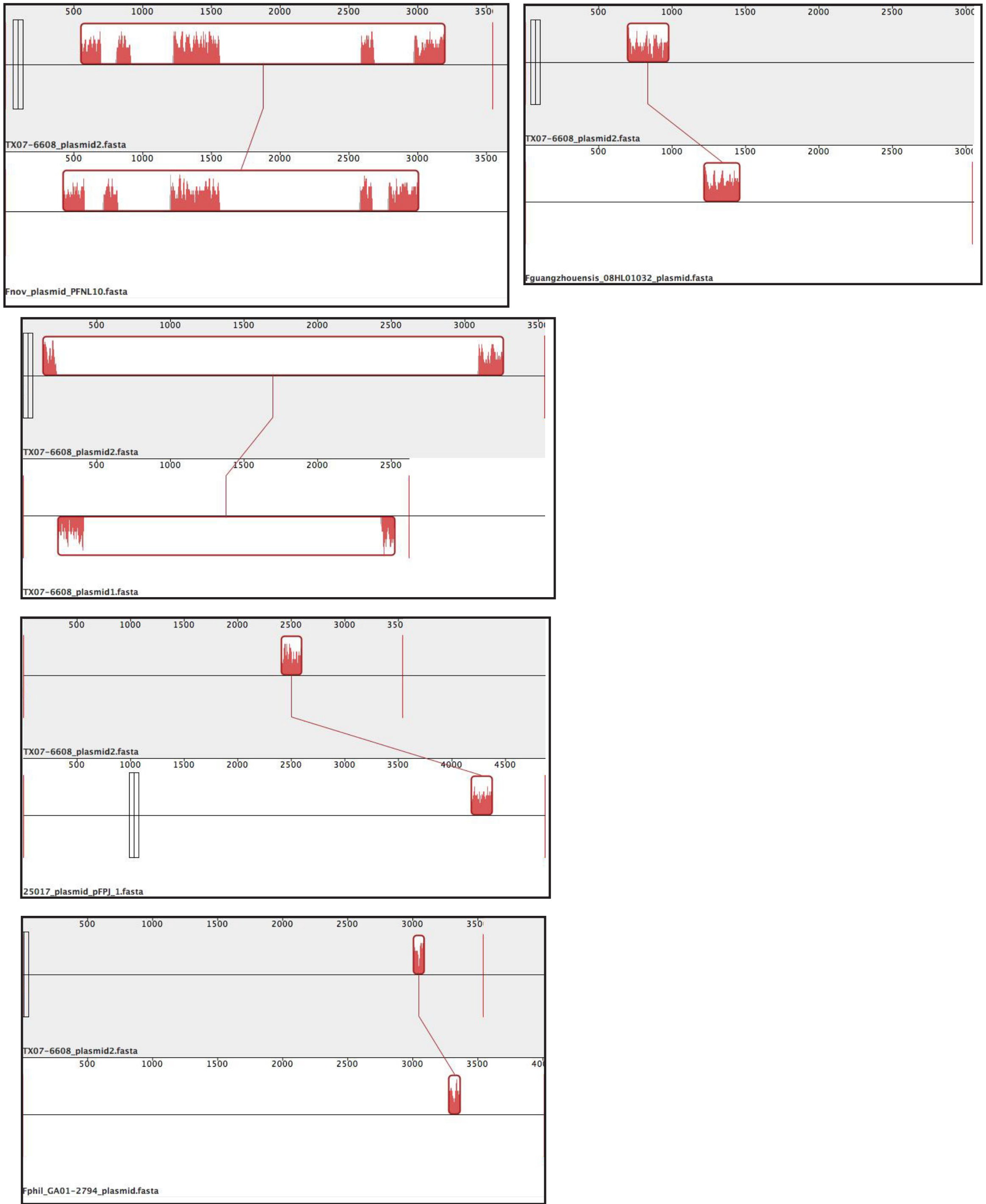

C. The plasmid from MA06-7296 had one small region of nucleotide sequence similarity to *F. philomiragia* plasmid pFPK_1.

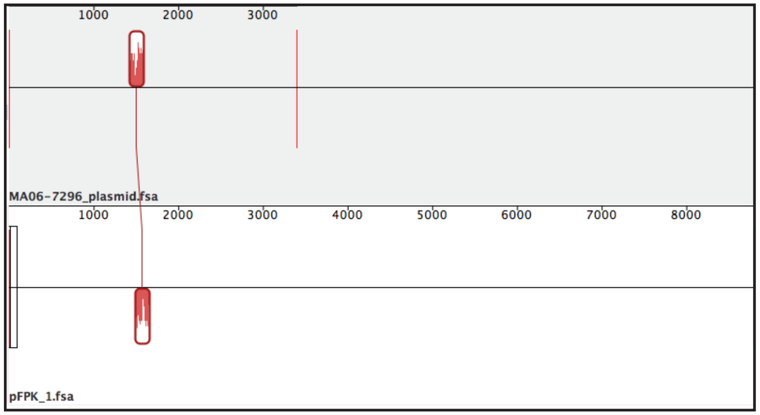

Supplement: S1 Fig — Regions of similarity in the comparisons are shown in green and red. (PDF) [file pone.0183554.s001.pdf]
