# Supplementary material for: Shared features of cryptic plasmids from environmental and pathogenic Francisella species
Source: PLoS One. 2017 Aug 24;12(8):e0183554. doi: 10.1371/journal.pone.0183554 (PMC5570271; doi:10.1371/journal.pone.0183554)

Figure S2. Progressive Mauve alignment of the AZ06-7470 plasmid with TX07-6608 plasmids 3 and 4, pFNPA10 from F. novicida-like strain PA10-7858, the DPG_3A-IS plasmid and the pFSC454 plasmid. The different regions of similarity are shown in different colors.

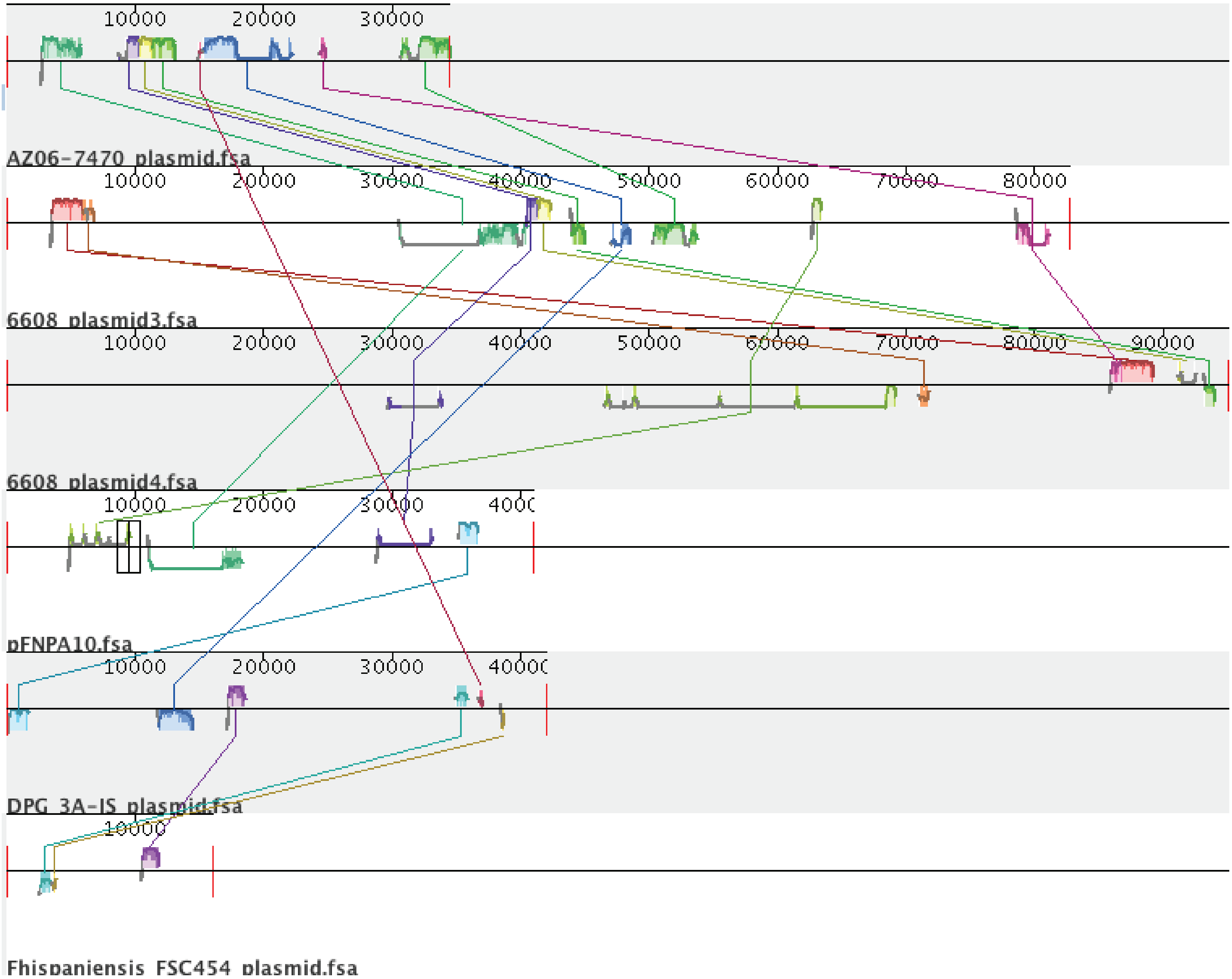

Supplement: S2 Fig — The different regions of similarity are shown in different colors. (PDF) [file pone.0183554.s002.pdf]
