# Supplementary material for: Shared features of cryptic plasmids from environmental and pathogenic Francisella species
Source: PLoS One. 2017 Aug 24;12(8):e0183554. doi: 10.1371/journal.pone.0183554 (PMC5570271; doi:10.1371/journal.pone.0183554)
Supplement: S3 Fig — The potential ori and ter regions are indicated by yellow vertical lines at the minimum and maximum GC skew values. Panel A. Plasmid 1. Panel B. Plasmid 2. Panel C. Plasmid 3. Panel D. Plasmid 4. (PDF) [file pone.0183554.s003.pdf]

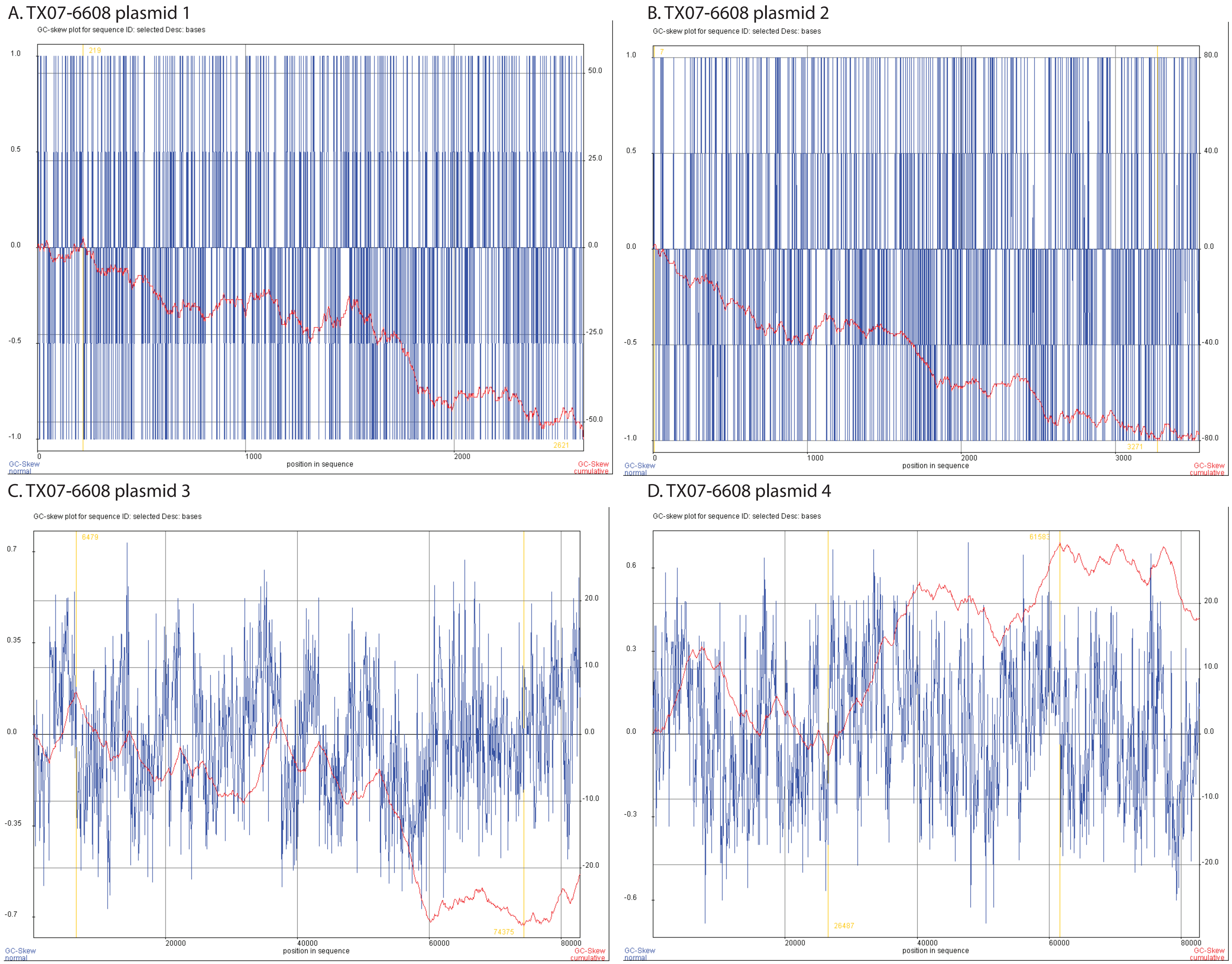
A. TX07-6608 plasmid 1
GC-skew plot for sequence ID: selected Desc: bases
219
2621
position in sequence
GC-Skew normal
GC-Skew cumulative
B. TX07-6608 plasmid 2
GC-skew plot for sequence ID: selected Desc: bases
7
3271
position in sequence
GC-Skew normal
GC-Skew cumulative
C. TX07-6608 plasmid 3
GC-skew plot for sequence ID: selected Desc: bases
6479
74375
position in sequence
GC-Skew normal
GC-Skew cumulative
D. TX07-6608 plasmid 4
GC-skew plot for sequence ID: selected Desc: bases
61583
26487
position in sequence
GC-Skew normal
GC-Skew cumulative
