# Supplementary figures and images for: Shared features of cryptic plasmids from environmental and pathogenic Francisella species
Source: PLoS One. 2017 Aug 24;12(8):e0183554. doi: 10.1371/journal.pone.0183554 (PMC5570271; doi:10.1371/journal.pone.0183554)

**A** AZ06-7470

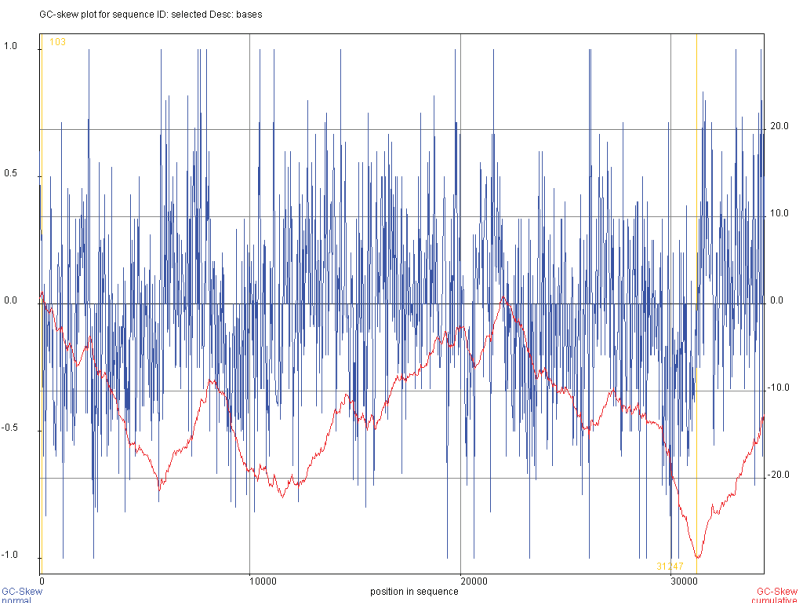

**B** CA97-1460

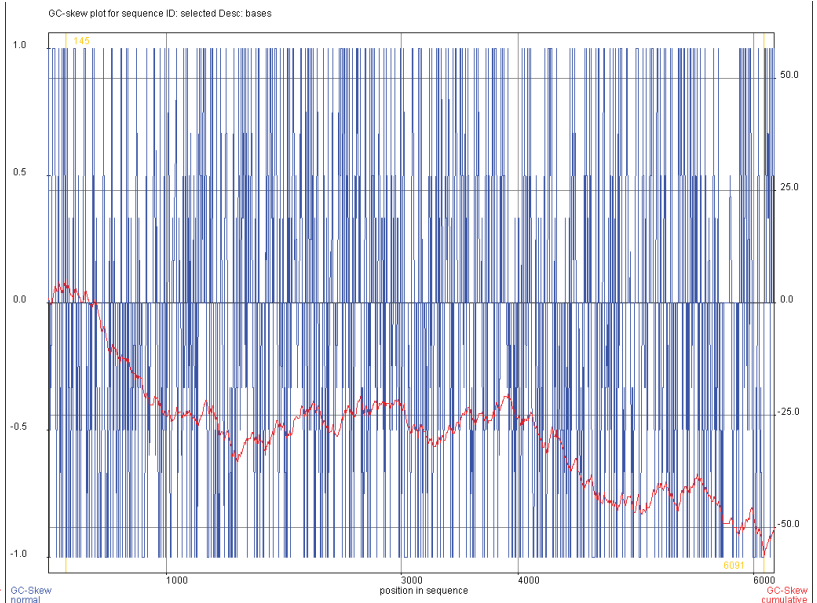

**C** MA06-7296

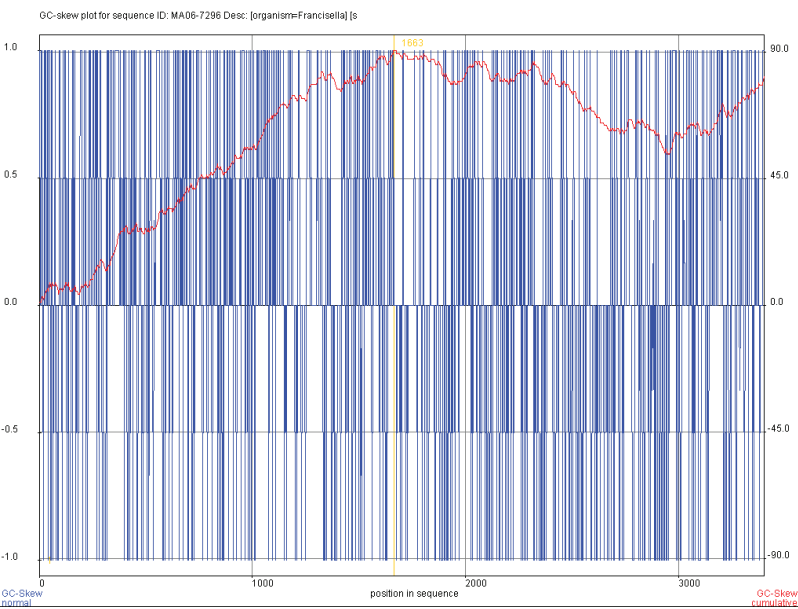

**D** DPG\_3A-IS

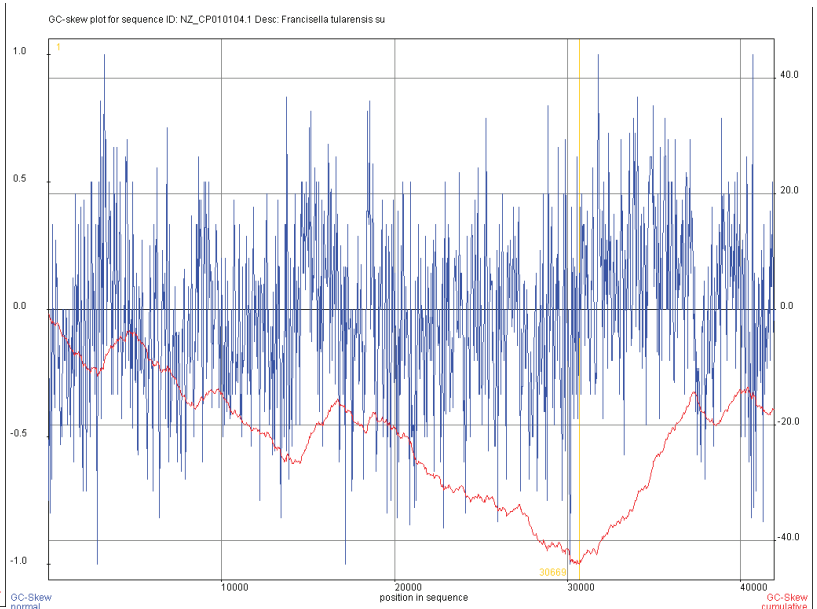

**E** FSC454

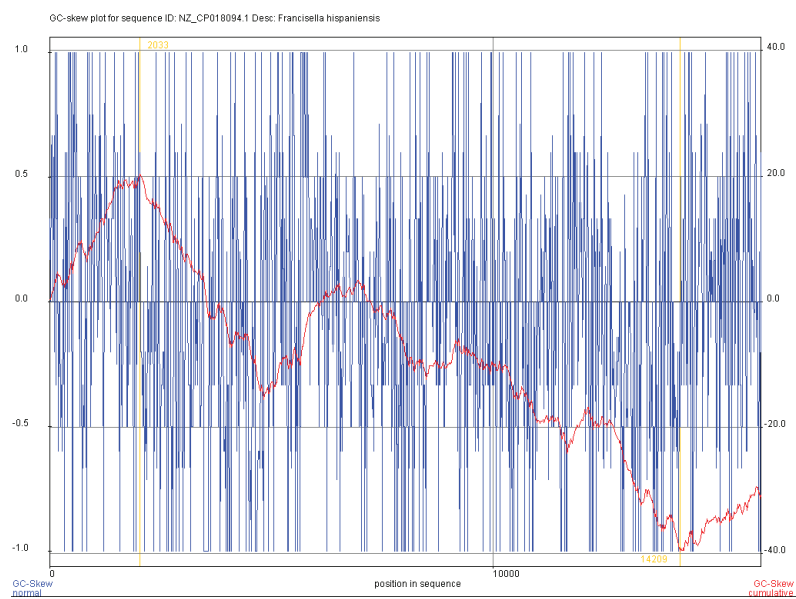

Supplement: S4 Fig — The potential ori and ter regions are indicated by yellow vertical lines at the minimum and maximum GC skew values. Panel A. AZ06-7470 plasmid. Panel B. CA97-1460 plasmid. Panel C. MA06-7296 plasmid. Panel D. DPG_3A-IS. Panel E. FSC454. The MA06-7296 plasmid did not have an ori region identified by this analysis, but the minimum GC skew value near 0. The DPG_3A-IS plasmid did not have a ter region identified by this analysis, by a maximum GC slew value occurred near 0. (PDF) [file pone.0183554.s004.pdf]
