# Supplementary material for: Shared features of cryptic plasmids from environmental and pathogenic Francisella species
Source: PLoS One. 2017 Aug 24;12(8):e0183554. doi: 10.1371/journal.pone.0183554 (PMC5570271; doi:10.1371/journal.pone.0183554)

Figure S5. Z-curves (AT, GC, RY and MK disparity curves) from OriFinder analysis of TX07-6608 plasmids 3 (Panel A) and 4 (Panel B), DPG_3A-IS plasmid (Panel C). Purple peaks with diamonds indicate the DnaA box clusters.

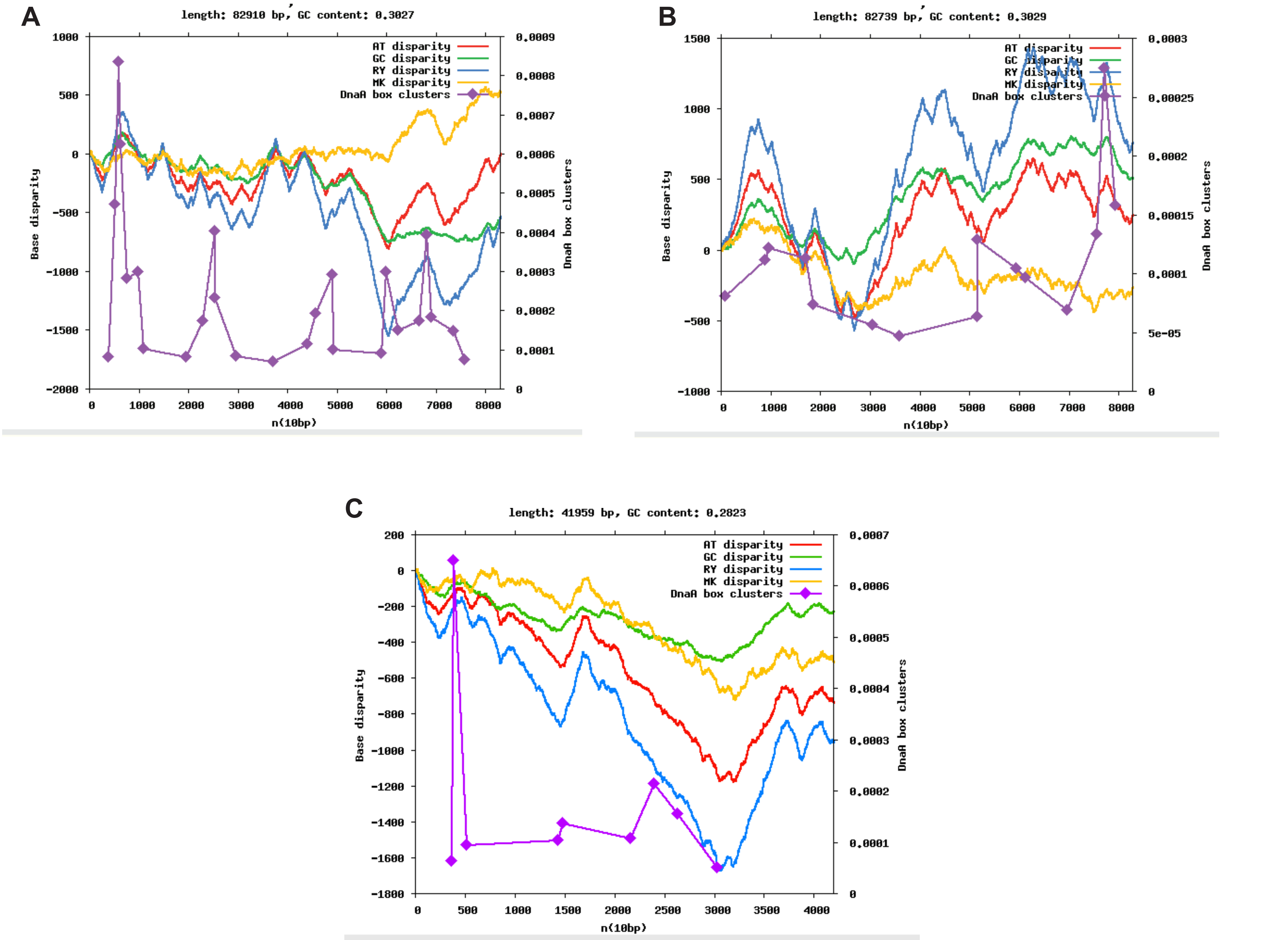

Supplement: S5 Fig — Purple peaks with diamonds indicate the DnaA box clusters. (PDF) [file pone.0183554.s005.pdf]
