# Supplementary material for: Shared features of cryptic plasmids from environmental and pathogenic Francisella species
Source: PLoS One. 2017 Aug 24;12(8):e0183554. doi: 10.1371/journal.pone.0183554 (PMC5570271; doi:10.1371/journal.pone.0183554)
Supplement: S1 Table — (PDF) [file pone.0183554.s006.pdf]

Table S1. Features of the *Francisella novicida-like* TX07-6608, *F. opportunistica* MA06-7296, *F. novicida* AZ06-7470 and *F. frigiditurris* CA97-1460 plasmids. The plasmids from *F. novicida* DPG_3A-IS and *F. hispaniensis* FSC454 are included for comparison purposes.

***Francisella* sp. TX07-6608**					
**Plasmid 1**	**2,621 bp**	**1 ORF**			
Locus_tag	ORF size (bp)	protein size (aa)	annotated function	closest database hit(s) (species, identifier, %identity)	hit location (chromosome, plasmid)
KK00_2307	926	308	replication family protein	*F. philomiragia* ATCC 25016, F242_0002, 29%;<br>*F. philomiragia* GA01-2801, LA02_2035, 30%;<br>*Allofrancisella guangzhouensis,* SD28_07730, 29%;<br>*F. philomiragia* GA01-2794, LA55_2099, 28%	pF242<br>pFPK_2<br>plasmid<br>plasmid
**Plasmid 2**	**3,546 bp**	**3 ORFs**			
Locus_tag	ORF size (bp)	protein size (aa)	annotated function	closest database hit(s) (species, identifier, %identity)	hit location (chromosome, plasmid)
KX00_2304	366	121	helix-turn-helix domain protein	*A. guangzhouensis*, SD28_06415, 68%	chromosome
KX00_2305	1,533	510	hypothetical protein	no hits	NA
KX00_2306	339	112	hypothetical protein	TX07-6608, KX00_2185, partial alignment 83%	plasmid 3
**Plasmid 3***	**82,910 bp**	**91 ORFs**			
Locus_tag	ORF size (bp)	protein size (aa)	annotated function	closest database hit(s) (species, identifier, %identity)	hit location (chromosome, plasmid)
KX00_2112	993	330	transposase	TX07-6608, KX00_228, 100%	plasmid 4
KX00_2113	324	107	transposase	TX07-6608, KX00_2282, 100%	plasmid 4
KX00_2115	1,776	591	group II intron reverse transcriptase	TX07-6608, KX00_2279/KX00_2277/ KX00_2239/KX00_2206, 96%	plasmid4
KX00_2117	363	120	DNA binding protein HU	TX07-6608, KX00_2208, 77%	plasmid 4<br>pFNPA10

KX00_2120	564	187	DNA-3-methyladenine glycosylase	*Francisella* sp. FSC1006, LO80_09240, 78%	chromosome
KX00_2122	504	167	single-stranded DNA-binding protein	*A. guangzhouensis,* SD28_01055, 56%	chromosome
KX00_2123	852	283	recombination-associated protein	*Francisella* sp. W12-1067, FRA_24c00970, 82%	ND
KX00_2128	603	200	transposase	TX07-6608 KX00_2214 69%	plasmid 4
KX00_2131	330	109	toxin RelE	*Leucothrix mucor*, WP_026744838.1, 64%	chromosome
KX00_2132	294	97	transcriptional regulator	*Psychromonas* sp. CNPT3, PCNPT3_06200, 51%	chromosome
KX00_2133	978	325	transposase	TX07-6608, KX00_2300 + many copies at 100%	plasmid 4
KX00_2135	2,364	787	conjugal transfer protein TraG	TX07-6608, KX00_2216, 85%	plasmid 4
KX00_2136	462	153	single-stranded DNA-binding protein	*F. novicida* PA10-7858, N894_0021 34%	pFNPA10
KX00_2138	453	150	transposase	*F. tularensis*, multiple genomes, 72%	chromosome
KX00_2139	120	39	transposase	TX07-6608, KX00_2259 + many copies at 100%	plasmid 4
KX00_2140	978	325	transposase	TX07-6608, KX00_2300 +many copies at 100%	plasmid 4
KX00_2141	237	78	plasmid pRiA4b ORF-3-like family protein	*Halobacteroides halobius* DSM 5150, Halha_1116, partial alignment 52%	chromosome
KX00_2142	1,236	411	group II intron reverse transcriptase	TX07-6608, KX00_2279 + 2 copies, 97%	plasmid 4
KX00_2147	459	152	conjugal transfer protein TraF	*F. novicida* PA10-7858, N894_0014, 30%	pFNPA10
KX00_2149	462	153	single-strand binding family protein	*F. novicida* PA10-7858, N894_0021, 33%	pFNPA10
KX00_2150	258	85	transposase	TX07-6608, KX00_2272, 100%	plasmid 4
KX00_2152	651	216	transposase	TX07-6608, KX00_2272, 93%	plasmid 4
KX00_2153	1,977	658	conjugal transfer protein TraG	TX07-6608, KX00_2216, 89%	plasmid 4

KX00_2157	1,236	411	group II intron reverse transcriptase	TX07-6608, KX00_2279 + 2 copies, 97%	plasmid 4
KX00_2159	561	186	site-specific recombinase?	*Legionella drancourtii* 62%<br>TX07-6608, KX00_2210, 35%; *F. novicida* PA10-7858, N894_0050, 38%	ND<br>plasmid 4; pFNPA10
KX00_2160	1,170	389	transposase	TX07-6608, KX00_2293, 42%; KX00_2294, 53%	plasmid 4
KX00_2161	3,012	1,003	transposase	TX07-6608, KX00_2280, KX00_2240, 100%	plasmid 4
KX00_2162	1,236	411	group II intron reverse transcriptase	TX07-6608, KX00_2279 + 2 copies, 97%	plasmid 4
KX00_2164	1,236	411	group II intron reverse transcriptase	TX07-6608, KX00_2278 + 2 copies, 57%	plasmid 4
KX00_2166	297	99	transposase	*F. tularensis,* multiple genomes, 87%	chromosome
KX00_2167	621	206	site-specific recombinase?	*Legionella drancourtii* 68%<br>*F. novicida* PA10-7858, N894_0001, 39%	ND<br>pFNPA10
KX00_2171	183	60	transposase	TX07-6608, KX00_2286 + many copies at 98%	plasmid 4
KX00_2172	603	200	transposase	TX07-6608 KX00_2272 99%	plasmid 4
KX00_2173	501	166	transposase	*F. philomiragia* 25017, Fphi_0511, 88%	chromosome
KX00_2174	1,842	613	SAM-dependent methyltransferase	*F. tularensis,* multiple genomes, 81%	chromosome
KX00_2175	1,023	340	transposase	TX07-6608, KX00_2272. 99%	plasmid 4
KX00_2177	4,275	1,424	conjugal transfer protein TraA	TX07-6608, KX00_2206;KX00_2301, 88%	plasmid 4
KX00_2180	555	184	sensory box protein	TX07-6608, KX00_229; KX00_2223, 53%	plasmid 4
KX00_2182	5,799	1,932	non-ribosomal peptide synthetase	*F. novicida* DPG_3A-IS, CH70_1995, 78%	plasmid
KX00_2183	963	320	ParB/RepB/Spo0J partition domain protein	Rickettsiales bacterium Ac37b, NOVO_09455, 38%<br>TX07-6608, KX00_2243, 26%	plasmid<br>plasmid 4
KX00_2184	1,014	337	ParA/ParB	*Legionella pneumophila*, 43%	ND<br>pFPK_1
KX00_2187	585	194	helix-turn-helix family protein	*Piscirickettsia salmonis*, KW89_2249, 28%	chromosome
KX00_2193	897	298	toxin co-regulated	*Fangia hongkongensis,* 35% partial alignment	ND

			pilus biosynthesis Q family protein		
KX00_2195	261	86	transposase	TX07-6608, KX00_2234 100%	plasmid 4
KX00_2196	231	76	helix-turn-helix domain protein	TX07-6608, KX00_2233 100%	plasmid 4
KX00_2197	978	325	transposase	TX07-6608, KX00_2300 + many copies at 100%	plasmid 4
KX00_2198	468	155	phosphotransferase enzyme family protein	*Holospora undulata,* K737_300648, 76%	ND
KX00_2199	333	110	antitoxin HicB	uncultured bacterium, ACD_21C00221G0001, 57%	ND
KX00_2201	699	232	transposase	TX07-6608, KX00_2235 + many copies at 100%	plasmid 4
**Plasmid 4***	**82,739 bp**	**102 ORFs**			
Locus_tag	ORF size (bp)	protein size (aa)	annotated function	closest database hit species, identifier, %identity	hit location (chromosome, plasmid)
KX00_2202	225	74	integrase	*Francisella* sp. W12-1067, FRA_44c11820, partial alignment 90%	ND
KX00_2204	180	59	transposase	TX07-6608, KX00_2197 + many copies, 68% first 59 aa align	plasmid 3
KX00_2205	978	325	transposase	TX07-6608 KX00_2197 + 2 copies, 100%	plasmid 3
KX00_2206	1,107	368	conjugal transfer protein TraA	TX07-6608, KX00_2177, 91%	plasmid 3
KX00_2208	366	121	DNA binding protein HU	TX07-6608, KX00_2117, 77%	plasmid 3
KX00_2210	621	206	site-specific recombinase	*Legionella fairfieldensis* 65%	ND
KX00_2212	1,233	410	DNA polymerase	*Fangia hongkongensis* 43%	ND
KX00_2213	654	217	transcriptional regulator, LexA-like or XRE family	*F. philomiragia* multiple genomes 65%	chromosome
KX00_2218	978	325	transposase	TX07-6608, KX00_2197 + 2 copies, 100%	plasmid 3
KX00_2220	231	76	transposase	TX07-6608, KX00_2160;KX00_2160, 53%	plasmid 3
KX00_2221	1,941	646	lipase	*F. novicida* GA99-3549, FTCG_01014, 36%; *F.*	chromosome

				novicida F6168, AS84_1001, 36%	
KX00_2223	1,143	380	sensory box protein	TX07-6608, KX00_2180, 53%	plasmid 3
KX00_2226	249	82	integrase	*Francisella* sp. W12-1067, many copies, partial alignment 89%	ND
KX00_2227	978	325	transposase	TX07-6608, KX00_2197 + 2 copies, 100%	plasmid 3
KX00_2228	978	325	transposase	TX07-6608, KX00_2197 + 2 copies, 100%	plasmid 3
KX00_2229	228	75	integrase	TX07-6608, KX00_2134, 100% partial alignment	plasmid 3
KX00_2230	435	144	integrase	*Francisella* sp. W12-1067, partial alignment 92%	ND
KX00_2231	996	331	RepB	*Francisella* sp. W12-1067, 49%	ND
				F. philomiragia ATCC25017, LA56_RS09235, 39% ; *F. novicida* F6168, 36%; F243_0006, 41%	pFPJ_1; pFNL10; pF243
KX00_2232	978	325	transposase	TX07-6608 KX00_2197 + 2 copies, 100%	plasmid 3
KX00_2233	975	324	integrase	TX07-6608, KX00_2196;KX00_2196, 100%	plasmid 3
KX00_2234	261	86	transposase	TX07-6608, KX00_2197 + 2 copies, 100%	plasmid 3
KX00_2235	243	80	transposase	TX07-6608, KX00_2201, 100%	plasmid 3
KX00_2237	231	76	transposase	TX07-6608, KX00_2201, 100%	plasmid 3
KX00_2239	1,236	411	group II intron reverse transcriptase	TX07-6608, KX00_2164 + 4 copies, 97%	plasmid 3
KX00_2240	276	91	transposase	TX07-6608, KX00_2161, 100%	plasmid 3
KX00_2241	978	325	transposase	TX07-6608, KX00_2197 + 2 copies, 100%	plasmid 3
KX00_2242	639	212	ParM	*Legionella shakespearei* 58%; *F. uliginis* TX07-7310, 40%	ND; chromosome
KX00_2243	867	288	ParB/RepB/Spo0J	*Legionella moravica,* 34%; TX07-6608, KX00_2183, 26%	ND plasmid 3
KX00_2248	282	93	DNA-binding protein HU	*F. novicida* PA10-7858, N894_0031, 81%	pFNPA10
KX00_2252	1,143	379	putative S-adenosylmethionine-dependent methyltransferase	*Fangia hongkongensis,* 61%	ND
KX00_2256	1,824	607	transposase	TX07-6608, KX00_2161, 22% partial alignment	plasmid 3
KX00_2258	978	325	transposase	TX07-6608, KX00_2197 + 2 copies, 100%	plasmid 3
KX00_2259	273	90	transposase	TX07-6608, KX00_2197 + 2 copies, 100%	plasmid 3

KX00_2261	978	325	transposase	TX07-6608, KX00_2197 + 2 copies, 100%	plasmid 3
KX00_2262	243	80	transposase	TX07-6608, KX00_2201, 98% aligns with end	plasmid 3
KX00_2264	978	325	transposase	TX07-6608, KX00_2197 + 2 copies, 100%	plasmid 3
KX00_2265	522	173	Pyridoxamine 5'-phosphate oxidase	*Legionella sainthelensi* 48%<br>TX07-6608, KX00_2131, 35%	ND<br>plasmid 3
KX00_2266	1,044	347	RepB	*F. novicida* PA10-7858, N894_0057. 50%	pFNPA10
KX00_2267	573	190	integrase	*Candidatus Competibacter denitrificans,* 65%	ND
KX00_2270	309	102	transcriptional regulator, Cro/CI/xre family	*Legionella hackeliae* 56%	chromosome
KX00_2271	558	185	transposase	TX07-6608, KX00_2197 + 2 copies, 100%	plasmid 3
KX00_2272	1,023	340	transposase	TX07-6608, KX00_2175, 95%	plasmid 3
KX00_2273	546	181	transposase	TX07-6608 KX00_2197 + 2 copies, 100%	plasmid 3
KX00_2275	231	76	transposase	TX07-6608, KX00_2201, 100% aligns with first 73 aa	plasmid 3
KX00_2277	1,236	411	group II intron reverse transcriptase	TX07-6608, KX00_2164 + 4 copies, 97%	plasmid 3
KX00_2279	1,236	411	group II intron reverse transcriptase	TX07-6608, KX00_2164 + 4 copies, 97%	plasmid 3
KX00_2280	276	91	transposase	TX07-6608, KX00_2161, 100%	plasmid 3
KX00_2281	429	142	transposase	TX07-6608, KX00_2197 + 2 copies, 100%	plasmid 3
KX00_2282	429	142	transposase	TX07-6608, KX00_2112, 100%	plasmid 3
KX00_2283	561	186	transposase	TX07-6608 KX00_2197 + 2 copies, 100%	plasmid 3
KX00_2284	978	325	transposase	TX07-6608 KX00_2197 + 2 copies, 100%	plasmid 3
KX00_2285	468	155	Initiator replication protein	*F. philomiragia* ATCC25017, F243_0006, 40% partial	pF243
KX00_2286	978	325	transposase	TX07-6608 KX00_2197 + 2 copies, 100%	plasmid 3
KX00_2290	255	84	integrase	TX07-6608, KX00_2160, 41%	plasmid 3
KX00_2291	324	107	Initiator replication protein	*Francisella* sp. W12-1067, FRA_71c15850, 50% partial	
KX00_2292	252	83	integrase	TX07-6608, KX00_2160, 53% partial alignment	plasmid 3
KX00_2293	786	261	integrase	TX07-6608, KX00_2160, 41%	plasmid 3

KX00_2294	231	76	transposase	TX07-6608, KX00_2160, 53% partial alignment	plasmid 3
KX00_2295	1,941	646	lipase	*F. novicida* GA99-3549, FTCG_01014, 36%	chromosome
KX00_2297	1,143	380	Signal transduction histidine kinase	TX07-6608, KX00_2180, 53%	plasmid 3
KX00_2300	978	325	transposase	TX07-6608, KX00_2197 + 2 copies, 100%	plasmid 3
KX00_2303	342	113	transposase	TX07-6608, KX00_2197 + 2 copies, 100%	plasmid 3
***F. opportunistica* MA06-7296**					
**Plasmid 1**	**3,403 bp**	**5 ORFs**			
Locus_tag	ORF size (bp)	protein size (aa)	annotated function	closest database hit species, identifier, %identity	hit location (chromosome, plasmid)
BBG19_1748	1,107	368	mobilization protein/plasmid recombination enzyme	*Clostridium botulinum*, WP_061309174.1, 63%	ND
BBG19_1749	348	115	hypothetical protein	no hits	
BBG19_1750	183	60	hypothetical protein	no hits	
BBG19_1751	177	58	hypothetical protein	no hits	
BBG19_1752	459	152	hypothetical protein	*Clostridium botulinum*, WP_053532535.1, 94%	ND
***F. novicida* AZ06-7470**					
**pFNE_1***	**34,471 bp**	**52 ORFs**			
Locus_tag	ORF size (bp)	protein size (aa)	annotated function	closest database hit species, identifier, %identity	hit location (chromosome, plasmid)
KX02_1830	147	48	initiator replication protein	TX07-6608, KX00_2266, 100% last 48 aa	plasmid 4
KX02_1831	384	127	thioesterase	*F. frigiditurris* CA97-1460, KX01_303, 90% partial alignment	chromosome

KX02_1833	579	192	XRE family transcriptional regulator	*Piscirickettsia salmonis*, KW89_3p87, 30%	pPSA1-3
KX02_1840	1,140	379	restriction-modification methylase	TX07-6608, KX00_2252, 97%	plasmid 4
KX02_1841	387	128	transposase	*Rickettsia* endosymbiont of *Ixodes pacificus*, REIP_0821, 56%	ND
KX02_1842	285	94	integrase	*Candidatus Caedibacter acanthamoebae*, M40_09375, 72%	plasmid
KX02_1843	288	95	integrase	*Rickettsia* endosymbiont of *Ixodes pacificua*, REIP_1487, 84%	ND
KX02_1846	225	74	transposase	*F. tularensis*, *F. opportunistica* MA06-7296, BBG19_0173, 100% partial alignment	chromosome
KX02_1848	3,024	1,007	transposase	TX07-6608, KX00_2256, 97% partial alignment	plasmid 4
KX02_1849	576	191	resolvase	Tx07-6608, KX00_2126, 93%	plasmid 3
KX02_1850	948	315	merR regulatory family protein	*Fangia hongkongensis*, WP_018298709.1, 54%	ND
KX02_1852	141	46	putative transposase	*Francisella* sp. W12-1067, FRA_44c12930, 91%	ND
KX02_1853	564	187	transposase	TX07-6608, KX00_2159, 88% + other copies	plasmid 3
KX02_1854	2,634	877	transposase	*F. novicida* DPG_3A-IS, CH70_1118 91%	chromosome
KX02_1857	123	40	transposase	TX07-6608, KX00_2171, 90%	plasmid 3
KX02_1858	315	104	transposase	*Alteromonas lipolytica*, BFC17_07755, 70%	ND
KX02_1859	135	44	transposase	*Piscirickettsia salmonis*, AVI48_08835, 62%	chromosome
KX02_1862	480	159	integrase	*Legionella spiritensis*, Lspi_2204, 76%	ND
KX02_1868	195	64	transposase	*F. tularensis*, multiple genomes, 88%	chromosome
KX02_1872	114	37	integrase	*Paenibacillus terrae* HPL-003, HPL003_22625, 73%	chromosome
KX02_1876	660	219	chromosome partitioning protein ParA	*Nitrosomonas ureae*, SAMN05216406_13719, 46%	ND
KX02_1880	573	190	integrase	TX07-6608, KX00_2267, 96%	plasmid 4
ORF not called coordinates 33574-34470	1,071	356	Rep	TX07-6608, KX00_2266, 70%	plasmid 4

***F. frigiditurris* CA97-1460**					
**pFCD_1**	**6,176 bp**	**7 ORFs**			
Locus_tag	ORF size (bp)	protein size (aa)	annotated function	closest database hit species, identifier, %identity	hit location (chromosome, plasmid)
KX01_RS09210	291	96	hypothetical	*Sphingobium* sp. CCH11-B1, WP_066769463, 37%	ND
KX01_RS09215	276	91	hypothetical	*Alphaproteobacteria* bacterium 41-28, BGO67_01715, 49%	ND
KX01_RS09220	681	226	hypothetical	*Francisella* cf. tularensis subsp. 3523, FN3523_0715, 61%	chromosome
KX01_RS09225	1,857	618	MobB	*Francisella* cf. tularensis subsp. novicida 3523, FN3523_0716, 60%	chromosome
KX01_RS09230	552	183	hypothetical	no hits	NA
KX01_RS09235	276	91	hypothetical	no hits	NA
KX01_RS09240	828	275	RepB	TX07-6608, KX00_2266, 43%	plasmid 4
***F. novicida* DPG_3A-IS**					
**Plasmid 1***	**41,959 bp**	**42 ORFs**			
Locus_tag	ORF size (bp)	protein size (aa)	annotated function	closest database hit species, identifier, %identity	hit location (chromosome, plasmid)
CH70_RS09710	561	186	resolvase	*F. novicida* PA10-7858, N894_0050, 99%	pFNPA10
CH70_RS09715	360	119	transcriptional regulator	*F. novicida* PA10-7858, N894_0049, 85%	pFNPA10
CH70_RS09720	678	225	Fic protein family	*Fangia hongkongensis,* WP_018299747, 82%	ND
CH70_RS09725	393	pseudo	DDE transposase	NA	NA
CH70_RS09740	360	119	hypothetical protein	*Francisella cf. novicida* Fx1, FNFX1_0922, 89%	chromosome
CH70_RS09745	599+	pseudo	transposase	NA	NA
CH70_RS09750	993	330	IS5/IS1182 family transposase	*F. tularensis* subsp. novicida PA10-7858, N894_0399, 99%	chromosome
CH70_RS09760	885	294	response regulator	*F. tularensis*, multiple genomes, WP_032730214, 100%	chromosome
CH70_RS09765	873	290	transposase	TX07-6608, multiple copies, 75%	chromosome

CH70_RS09780	744	247	DDE transposase	*F. tularensis* subsp. novicida GA99-3548, FTDG_01089, 99% + others	chromosome
CH70_RS10200	128+	pseudo	transposase	NA	NA
CH70_RS09785	774	247	DDE transposase	*F. tularensis* subsp. novicida GA99-3548, FTCG_01150, 99% + others	chromosome
CH70_RS09790	2877	958	DDE transposase	mutiple Francisella genomes, 97%+	various
CH70_RS09795	555	184	transposase	*F. frigiditurris* CA97-1460, KX01_1492, 98%	chromosome
CH70_RS09805	978	325	cell division protein Fic	*F. frigiditurris* CA97-1460, KX01_1490, 100%	chromosome
CH70_RS09815	642	213	plasmid partitioning APTase ParM	*F. hispaniensis* FSC454, AUF42_00010, 97%	ND
CH70_RS09825	867	288	transposase	*F. uliginis* TX077310, F7310_07010, 93%	chromosome
CH70_RS09830	216	71	transposase	multiple *Francisella* genomes, 100%	various
CH70_RS09835	1161	386	two-component sensor histidine kinase	*F. uliginis* TX077310, F7310_05465, 52%	chromosome
CH70_RS09840	5814	1937	non-ribosomal peptide synthetase	TX07-6608, KX00_2182, 79%	plasmid 3
CH70_RS09845	873	290	transposase	TX07-6608, multiple hits, 75%	chromosome
CH70_RS09855	557+	pseudo	transposase	NA	NA
CH70_RS09865	744	247	DDE transposase	multiple *Francisella* genomes, 100%	various
CH70_RS09870	993	330	IS5/IS1182 family transposase	multiple *Francisella* genomes, 100%	various
CH70_RS09875	297	98	transposase	TX07-6608, KX00_2166, 88%	plasmid 3
CH70_RS09880	873	290	transposase	TX07-6608, multiple hits, 75%	various
CH70_RS09885	284+	pseudo	invertase	NA	NA
CH70_RS09890	1539	512	replication initiation protein	*F. tularensis* subsp. novicida FAI, DR83_1899, 98%	ND
CH70_RS09900	873	290	transposase	TX07-6608, multiple hits, 75%	various
CH70_RS09905	297	98	transposase	TX07-6608, KX00_2166, 88%	plasmid 3
CH70_RS09910	255	84	transcriptional regulator	*F. tularensis* subsp. novicida GA99-3548, FTDG_00231, 61%	ND
CH70_RS09915	333	110	mRNA interferase PemK	*Francisella* cf. novicida Fx1, FNFX1_0647, 64%	chromosome
CH70_RS09920	744	247	DDE transposase	multiple *Francisella* genomes, 100%	ND

CH70_RS09930	867	288	transposase	*F. tularensis* subsp. novicida FAI, multiple copies, 99%	
***F. hispaniensis* FSC454**					
**pFSC454***	**16,037 bp**	**13 ORFs**			
Locus_tag	ORF size (bp)	protein size (aa)	annotated function	closest database hit species, identifier, %identity	hit location (chromosome, plasmid)
FSC454_RS09425	729	242	hypothetical protein [response regulator]	*F. philomiragia* GA01-2801, LA02_1244, 39%	chromosome
FSC454_RS09435	1413	470	Rep	*F. novicida* PA10-7858, N894_0057, 43%	pFNPA10
FSC454_RS09440	294	97	transcriptional regulator	TX07-6608, KX00_2132, 97%	plasmid 3
FSC454_RS09445	330	109	addiction module toxin RelE	TX07-6608, KX00_2131, 96%	plasmid 3
FSC454_RS09455	666	221	IS5/IS1182 family transposase	*F. uliginis* TX077310, F7310_00920, 88%	chromosome
FSC454_RS09495	696	231	hypothetical protein [response regulator]	*F. philomiragia* GA01-2801, LA02_1244, 39%	chromosome
FSC454_RS09505	149+	pseudo	IS5 family transposase	NA	NA

*hypothetical not included
+pseudo genes with no stop or no start, ORF could not be determined
ND: replicons were not determined in genome sequence
