# Supplementary material for: Shared features of cryptic plasmids from environmental and pathogenic Francisella species
Source: PLoS One. 2017 Aug 24;12(8):e0183554. doi: 10.1371/journal.pone.0183554 (PMC5570271; doi:10.1371/journal.pone.0183554)
Supplement: S2 Table — (PDF) [file pone.0183554.s007.pdf]

Table S2. Tandem Repeats Finder output

	Indices	Period Size	Copy Number	Consensus Size	Percent Matches	Percent Indels	Score	A	C	G	T	Entropy (0-2)
6608 plasmid2	1630--1700	12	5.4	12	69	18	52	16	15	11	56	1.67
6608 plasmid3	11201--11262	12	5.2	12	100	0	124	24	24	0	51	1.48
	35292--35353	12	5.2	12	100	0	124	51	0	24	24	1.48
6608 plasmid4	11473--11512	13	3.0	14	75	24	50	35	0	7	57	1.27
	46997--47037	20	2.0	21	90	4	66	24	0	7	68	1.15
AZ06-7470 plasmid	29975--30023	8	6.1	8	100	0	98	24	24	12	38	1.90
	31065--31185	8	15.1	8	100	0	242	24	24	13	37	1.91
DPG_3A-IS plasmid	18311--18349	18	2.2	18	90	9	62	33	7	10	48	1.66
	20916--20946	9	3.4	9	100	0	62	22	0	9	67	1.19
pFSC454	2689--2726	18	2.1	19	85	5	51	36	5	7	50	1.54

Columns are:

Indices of the repeat relative to the start of the sequence.
Period size of the repeat.
Number of copies aligned with the consensus pattern.
Size of consensus pattern (may differ slightly from the period size).
Percent of matches between adjacent copies overall.
Percent of indels between adjacent copies overall.
Alignment score
Percent composition for each of the four nucleotides.
Entropy measure based on percent composition.
